# Ru(II)-Fenamic-Based Complexes as Promising Human Ovarian Antitumor Agents: DNA Interaction, Cellular Uptake, and Three-Dimensional Spheroid Models

**DOI:** 10.1021/acs.inorgchem.4c04344

**Published:** 2025-02-18

**Authors:** Tamara Teixeira, Marcos V. Palmeira-Mello, Pedro Henrique Machado, Carlos A. F. Moraes, Camila Pinto, Rayane C. Costa, Wladimir Badaró, José A. Gomes Neto, Javier Ellena, Fillipe Vieira Rocha, Alzir A. Batista, Rodrigo S. Correa

**Affiliations:** † Department of Chemistry, Institute of Exact and Biological Sciences, Federal University of Ouro Preto (UFOP), 35402-136 Ouro Preto, Minas Gerais, Brazil; ‡ Department of Chemistry, Federal University of São Carlos (UFSCar), 13565-905 São Carlos, São Paulo, Brazil; § Institute of Physics of São Carlos, University of São Paulo (IFSC/USP), 13566-590 São Carlos, São Paulo, Brazil; ∥ Institute of Chemistry, São Paulo State University (UNESP), 14800-900 Araraquara, São Paulo, Brazil

## Abstract

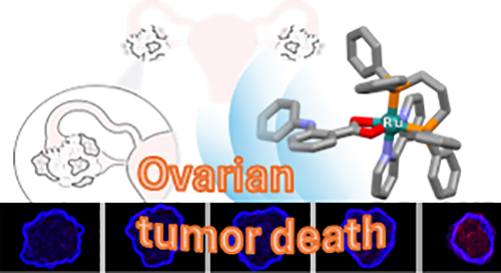

Cancer resistance to chemotherapeutic agents such as cisplatin presents a significant challenge, leading to treatment failure and poor outcomes. Novel metal-based compounds offer a promising strategy to overcome drug resistance and to enhance efficacy. Four Ru(II) complexes with fenamic acid derivatives were synthesized and characterized: [Ru(L)(bipy)(dppp)]PF_6_, where L represents fenamic acid (HFen, complex **1**), mefenamic acid (HMFen, complex **2**), tolfenamic acid (HTFen, complex **3**), and flufenamic acid (HFFen, complex **4**). Their composition was supported by molar conductivity, elemental analysis, Fourier transform infrared spectroscopy, ultraviolet–visible spectroscopy, mass spectrometry, and ^31^P{^1^H}, ^1^H, and ^13^C nuclear magnetic resonance, with the crystal structure of complex **1** confirmed via X-ray diffraction. Complexes **1**–**4** exhibited notable cytotoxicity against tested cell lines, particularly A2780 and A2780cisR (cisplatin-resistant ovarian tumors), compared to MDA-MB-231 (breast) and A549 (lung) lines. Mechanistic studies revealed weak DNA interactions through minor grooves or electrostatic binding. Cellular uptake assays showed effective internalization of complexes **1** (3.6%) and **2** (4.5%), correlating with potent IC_50_ values. These complexes also altered cell morphology, reduced cell density, and inhibited colony formation in the A2780 cells. Staining assays indicated induced cell death and organelle damage, highlighting their potential as promising antitumor agents.

## Abstract 2

Ruthenium(II) with fenamate derivatives exhibited enhanced activity against ovarian cancer cell lines, including cisplatin-resistant strains, compared to free ligands, a Ru(II) precursor, and cisplatin. The compounds weakly interact with DNA, penetrate cells as indicated by uptake studies, and exhibit notable activity in three-dimensional spheroid models. These findings highlight their potential as promising candidates for developing metallodrugs in cancer therapy.

## Introduction

Cell culture is fundamental for maintaining cells in the laboratory and has been crucial to significant scientific advances, particularly in the study of tumor cell biology.^1^ In two-dimensional (2D) experiments, cells are cultured as a monolayer on a flat surface, a method that has been critical in advancing drug development.^2^ However, discrepancies often arise between results obtained from *in vitro* and *in vivo* experiments due to differences in cellular behavior, including cell morphology, growth rate, oxygen gradients, and cell–cell interactions.^3,4^ To address these limitations, a three-dimensional (3D) cell culture has been developed, providing a more physiologically relevant approach that brings experimental conditions closer to the *in vivo* tumor microenvironment.^5^ In this context, 3D models more accurately represent cells and biological tissues, enhancing our understanding of biological processes and improving the evaluation of new treatments.^6^ By mimicking the complex environment found in living organisms, 3D cultures offer a more reliable platform for studying cellular behavior, drug responses, and disease progression than traditional 2D cultures.^7−10^

The clinical success of cisplatin and its analogues has attracted a significant amount of interest in exploring metal centers for the design of novel therapeutic agents. Despite the significant use of these complexes in cancer treatment, some problems related to resistance and side effects have resulted in researchers exploring alternative metals to platinum to get around these issues.^11,12^ Thus, ruthenium has been widely used, and a series of studies have shown that complexes based on this metal are very promising in the search for potential metallodrugs.^13−17^ It is important to mention that some ruthenium compounds have already progressed to clinical trials. An example through photodynamic therapy (PDT) includes the TLD1433 complex that recently has reached a Phase Ib human clinical trial (ClinicalTrials.gov; identifier, NCT03053635),^18,19^ and now Phase II is ongoing to evaluate the compound in 125 patients with BCG-intolerant NMIBC or BCG-unresponsive for treatment of bladder cancer (ClinicalTrials.gov; identifier, NCT03945162).^20^ Thus, Ru(II) complexes containing polypyridyl ligands have received widespread attention, mainly, as photosensitizers for photodynamic therapy.^21,22^

Also, a widely used strategy in the development of new metallodrugs is the use of chelating ligands derived from bioactive molecules to form stable complexes, thereby enhancing the biological activity of the resulting compounds. Coordination of bioactive ligands with metal ions can lead to improved pharmacokinetics, increased target specificity, and the ability to overcome drug resistance.^23^ In addition, the metal center often introduces unique redox, catalytic, or structural properties that further contribute to the therapeutic potential of the complexes. This strategy not only enhances the inherent bioactivity of the ligand but also enables the design of multifunctional drugs with tailored properties for specific medical applications.^24^ An interesting class of ligands for ruthenium involves fenamic derivatives, nonsteroidal anti-inflammatory drugs (NSAIDs), widely used to treat pains and inflammatory processes due to their analgesic, anti-inflammatory, and antipyretic properties.^25,26^ In addition, these compounds have potential in treating other diseases, such as cancer, due to their ability to form reactive metabolites via oxidation reactions.^27^ Recent studies suggest that the complexation of these molecules with metals represents a promising strategy in the search for new metallodrugs.^28−31^

Therefore, we describe here the synthesis of four ruthenium complexes with derivatives of fenamic acid as well as bipy (2,2′-bipyridine) and dppp [1,3′-bis(diphenylphosphine)propane] as ancillary ligands. The complexes were characterized by several techniques, presenting [Ru(L)(bipy)(dppp)]PF_6_ as the general formula [where L = fenamic acid (HFen, complex **1**), mefenamic acid (HMFen, complex **2**), tolfenamic acid (HTFen, complex **3**), and flufenamic acid (HFFen, complex **4**), bipy = 2,2′-bipyridine, and dppp = 1,3-bis(diphenylphosphino)propane], evaluated in terms of their effect on several cancer and normal cell lines, and evaluated in terms of their possible interactions with the DNA biomolecule. In addition, complexes **1** and **2** were selected for detailed studies to better understand their behavior in the ovarian tumor cell line (A2780), using a morphological model, colony formation, and cellular uptake evaluation. In addition, complex **1** was evaluated by using a 3D cell culture method to improve the predictability of its potential anticancer activity against the A2780 tumor line. This approach allows a more accurate assessment of the efficacy of the complex in a biological environment, providing deeper insight into its therapeutic viability.

## Results and Discussion

### Synthesis and Characterization

**Scheme 1:**
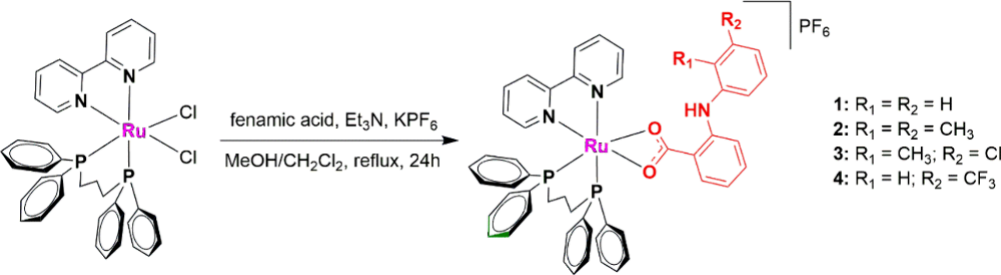
Synthetic Procedures for Complexes **1**–**4**

Ruthenium complexes containing fenamic acid derivatives were synthesized from the *cis*-[RuCl_2_(bipy)(dppp)] precursor by exchanging the two chlorido ligands with the carboxylate group, which coordinates in a bidentate form. All syntheses were carried out under reflux, and KPF_6_ was used as the counterion (see Scheme 1). The complexes obtained are soluble in dichloromethane, chloroform, acetone, and DMSO. The mass spectra of the complexes were obtained by ESI-QTOF-MS in the positive mode, and the corresponding data are illustrated in Figures S3–S6. The peaks related to the molecular ions of the complexes were accordingly observed with errors of <2 ppm (Table S1), confirming the proposed structures.

The molar conductivity of **1**–**4** indicated the presence of 1:1 electrolyte, according to the values reported in the literature (20–62 Ω^–1^ cm^2^ mol^–1^, in DMSO).^32^ The Fourier transform infrared (FT-IR) data of **1**–**4** exhibited a characteristic band corresponding to the ν(N–H) vibrations around 3300 cm^–1^, which was attributed to the fenamate ligand. This band is also present in the spectra of complexes **1**–**4**, suggesting that the ligands did not coordinate to the metal via the NH group (see the Supporting Information). The characteristic bands of the asymmetric (ν_a_COO^–^) and symmetric (ν_s_COO^–^) stretches of the carboxylate anion were observed at ∼1654 and ∼1434 cm^–1^, respectively, for the free ligands. The spectra of the complexes showed a shift in the asymmetric stretching to lower-energy regions compared to those of the free ligand. As a result of coordination of the fenamate ligand, there is a decrease in the character of the C=O bond. On the contrary, the symmetrical stretches appear at higher-frequency regions than in the free ligand because the double bond is delocalized after the coordination of the ligand. The electronic spectra of complexes **1**–**4** in the ultraviolet–visible (UV–vis) region showed bands at ∼295 nm, which can be attributed to the π → π* intraligand transitions. The band at ∼360 nm is attributed to metal ligand charge transfer (MLCT), involving the dπ_Ru_ → 3pσ*dπ_(dppp)_ and dπ_Ru_ → π*_(bipyridine and fenamic ligands)_ transitions, as reported in the literature for Ru(II)/phosphinic/diiminic complexes.^33^

The ^1^H nuclear magnetic resonance (NMR) spectra of the complexes exhibited overlapping signals corresponding to the aromatic protons of dppp, bipy, and fenamate ligands in the region of 8.70–6.04 ppm. A singlet signal at 9.00 ppm is characteristic of the N–H group in the fenamic ligands. The region around 3.00–1.89 ppm corresponds to the hydrogen atoms of the aliphatic carbon in the diphosphine, as well as the hydrogen atoms of the methyl groups in the mefenamic and tolfenamic ligands. The ^13^C{^1^H} NMR spectra of complexes **1**–**4** show a signal at 185 ppm, which refers to the quaternary carbon of the carboxylic group in the fenamic derivatives. Overall, the carbon signals are found in three regions. The signals between δ 30 and 14 ppm refer to the aliphatic carbons of the diphosphine ligand dppp and the methyl carbons of the mefenamic and tolfenamic ligands. The signals for the phenyl carbons of dppp, the fenamic ligands, and the bipyridine ligand are assigned to the region between δ 116 and 148 ppm, while the signals for some of the carbons bonded to nitrogen and to the quaternary carbon are attributed in the region above δ 148 ppm. Furthermore, in a recent study conducted by our group,^34^ a detailed and precise assignment of the ^1^H and ^13^C NMR signals was carried out for complexes analogous to those described herein. The results are consistent with the presented data.

The ^31^P{^1^H} NMR spectrum of the *cis*-[RuCl_2_(bipy)(dppp)] precursor shows two doublets at δ 38.32 and 30.33 ppm, related to the non-equivalence of the phosphorus atoms in the dppp ligand. This indicates that each phosphorus is *trans* to different atoms, in which one phosphorus atom is *trans* to the nitrogen atom of bipy and other is *trans* to the chlorido ligand, resulting in the *cis* isomer. The complexes synthesized with the fenamic ligands, based on the precursor *cis*-[RuCl_2_(bipy)(dppp)], also showed two doublets, demonstrating that the phosphorus atoms were neither chemically nor magnetically equivalent. As observed in the spectra of the new complexes, signal shifts were found in higher-frequency regions compared to those of the precursor because the carboxylate of the fenamic ligand acts as a weaker electron donor than the chlorido ligands (good σ- and π-donor).

Furthermore, the stability of the complexes was studied via ^31^P{^1^H} NMR in DMSO and a DMSO/DMEM (Dulbecco’s modified Eagle’s medium) solution over 48 h. All compounds exhibited stability in DMSO, whereas in the DMSO/DMEM solution, compounds **1**–**4** were less stable, presenting new signals in their ^31^P{^1^H} NMR spectra after 48 h (see the Supporting Information). In the same way, stability assays were performed for the precursor *cis-*[RuCl_2_(bipy)(dppp)]. In DMSO, new signals are observed at ∼40 ppm in the ^31^P{^1^H} NMR spectrum that can be attributed to the coordination of DMSO, exchanging the chlorido ligands. Also, the spectrum of the precursor complex in the culture medium showed, in addition to the signals related to DMSO coordination, two other doublets with chemical shifts at ∼33 and ∼46 ppm related to the coordination of another ligand species present in the culture medium, probably a carboxylate group due to the similarity with the chemical shifts of complexes **1**–**4**.

**Figure 1:**
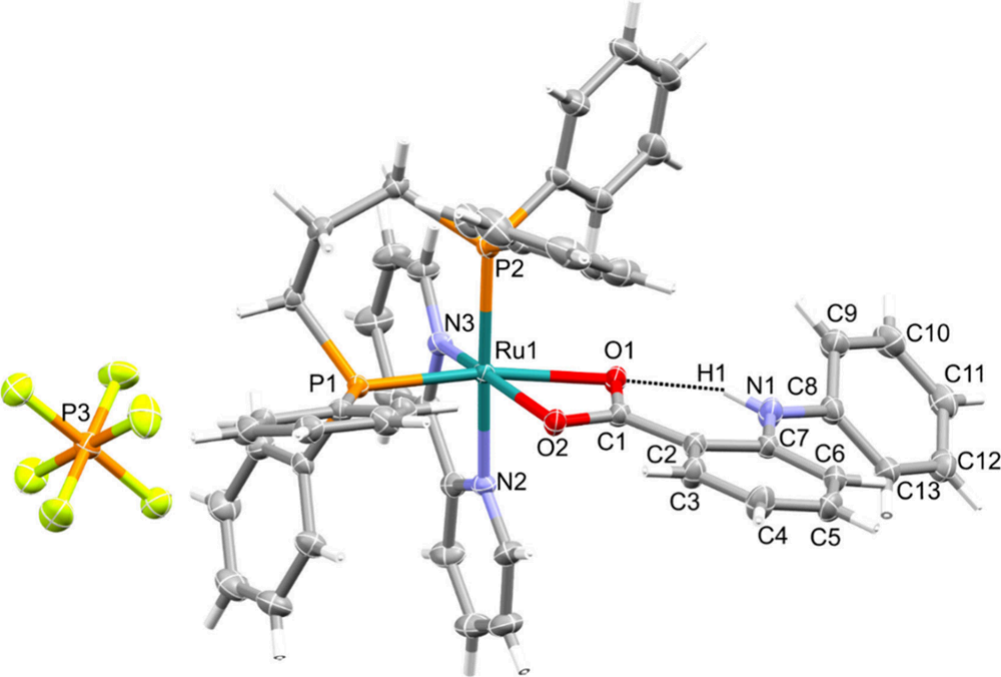
Crystallographic structure of complex **1** and the coordinated atoms and ruthenium labeled, with ellipsoids drawn at the 30% thermal probability level.

In addition, the crystal structure of complex **1** was confirmed by single-crystal X-ray diffraction (SCXRD) analysis (Figure 1). As expected, the complex presents a distorted octahedral geometry, with bond angles deviating from 90°. The stereochemistry of the compound has been confirmed. The fenamate ligand coordinates in a monoanionic bidentate mode by the O1 and O2 atoms of the carboxylate. As shown in Figure 1, the O1 atom is *trans* to the P1 atom of dppp while the O2 atom is *trans* to the N3 atom of bipy. The Ru1–O1 distance (2.226 Å) is longer than the Ru1–O2 bond (2.130 Å), which can be attributed to the stronger *trans* influence of the P1 phosphorus atom *trans* to the O1 atom, when compared to the *trans* influence of nitrogen (N3) that is *trans* to the O2 atom. The crystallographic parameters for the Ru–P (2.2424–2.3131 Å), Ru–N (2.068–2.111 Å), and Ru–O (2.130–2.226 Å) bond distances are in the range expected for Ru(II)/phosphinic complexes reported in the literature.^35−38^ The main crystallographic data and selected distances and bond angles are presented in Tables S2 and S3.

As illustrated in Figure 1, there is an intramolecular hydrogen bond (N1–H1···O1) with a separation of 1.955 Å. This interaction contributes to stabilization of the conformation of the fenamate ligand. Also, it may justify the broad infrared band related to the N1–H1 stretching vibration at ∼3300 cm^–1^ for complex **1**. The same band profile is observed for **2**–**4**, suggesting that this interaction could be present in these complexes.

After Ru(II) coordination, the fenamic ligand conformation changed slightly compared with that of the free ligand reported by Zhou et al.^39^ Also, in the free ligand, the C1=O1 bond length is 1.233 Å, while the C1–O2 bond length is 1.316 Å, which highlight the double- and single-bond character, respectively, of the carboxylic acid group. On the contrary, in complex **1**, the C1⋯O1 and C1⋯O2 bond lengths are 1.278 and 1.269 Å, respectively. Both values are comparable and fall between those expected for double and single bonds. This indicates that the coordinated carboxylate exhibits resonance and electronic delocalization in the O1⋯C1⋯O2 bonds. In the free ligand, the dihedral angle between the two aromatic rings is 71.39°; meanwhile, in the coordinated ligand, the dihedral angle is 35.08°. Additionally, an intramolecular π···π interaction is observed between the phenyl group of dppp and the aromatic ring of bipy, with a centroid separation of 3.658 Å. Furthermore, the crystal self-assembly is stabilized primarily by weak C–H···F and C–H···π interactions.

### DNA Interaction Studies

**Figure 2:**
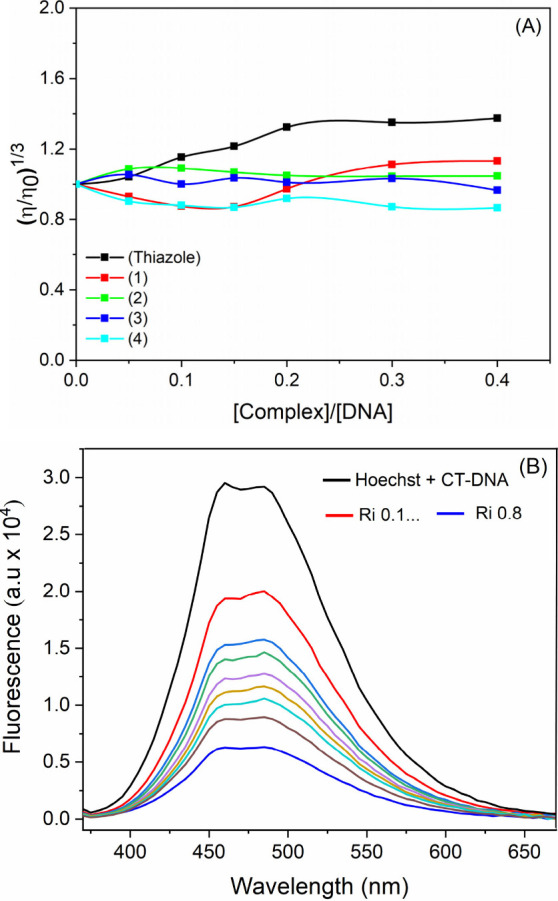
Interaction of complexes **1**–**4** with DNA. (A) Relative viscosity of CT-DNA after the addition of different concentrations of complexes **1**–**4** and thiazole orange at 25 °C. (B) Fluorescence emission spectra of DNA–Hoechst without the complex and with increasing amounts of complexes **1**–**4** (λ_exc_ = 343 nm).

DNA represents one of the targets for metal-based compounds.^40^ To obtain better insights regarding the DNA-interacting properties of **1–4**, viscosity, circular dichroism, and fluorescence competitive assays were performed using calf thymus DNA (CT-DNA). CT-DNA is a high-quality template double-stranded DNA form isolated from thymus calves. It has been employed for DNA polymerase assays, determination of DNA content in gels, and quantification by fluorescence experiments. Initially, the DNA concentration was measured using a spectrophotometer at 260 nm, based on the nucleobase molar absorptivity of 6600 M^–1^ cm^–1^. The viscosity results shown in Figure 2A indicate no significant variations in the relative viscosity of DNA [(η/η_0_)^1/3^], suggesting that complexes **1**–**4** interact with the biomolecule primarily through weak contacts. In contrast, positive controls, such as thiazole orange and cisplatin, demonstrate distinct mechanisms of interaction. Thiazole orange intercalates into DNA, causing the DNA duplex to lengthen,^41,42^ while cisplatin forms covalent bonds that distort and shorten the DNA duplex structure.^43^

The effect of these compounds on the DNA structure was investigated by circular dichroism (CD). CD is a spectroscopic technique that is useful for obtaining structural information about chiral biomolecules.^44^ The most commonly studied DNA, the B-form, exhibits positive and negative bands at 277 and 245 nm, respectively, which arise from base stacking and helicity properties. Our results revealed that in the presence of **1**–**4**, there are no significant changes in CD spectra, suggesting the absence of strong interactions, which leads to small changes in the secondary structure of the DNA macromolecule, as already reported for different metal-based compounds (Figure S27).^45,46^

Therefore, viscosity and dichroism studies indicate that only weak DNA interaction may occur with complexes **1**–**4**. Minor groove binding was assessed by a fluorescence competitive assay using Hoechst 33258. Hoechst is a probe that binds to DNA, emitting fluorescence at 460 nm. The displacement of this groove binding molecule by an external agent leads to fluorescence quenching. The spectral data obtained for **1**–**4** indicate that all compounds can displace the Hoechst, which was detected by decreasing fluorescence in the presence of these compounds (Figure 2B and Figure S28). Thus, according to the structures of the compounds, probably weak interaction involving minor grooves or electrostatics may be suggested.

### *In Vitro* Cytotoxicity

The cytotoxic activities of complexes **1**–**4**, the precursor complex, and the fenamic ligands were obtained against tumor cell lines A549, MDA-MB-231, A2780, and A2780cis and normal cell line MRC-5. For a comparative study, the cytotoxicity of cisplatin against these cell lines was also evaluated. The IC_50_ values and selectivity index (SI) are reported in Tables 1 and 2, respectively, and these results were obtained using the MTT method.

**Table 1:** *In Vitro* Cytotoxicity (IC_50_, micromolar) of Complexes **1–4**, [RuCl_2_(bipy)(dppp)], Free Ligands, and Cisplatin against Different Cell Lines

	MRC-5	A549	MDA-MB-231	A2780	A2780cis
**1**	2.47 ± 0.18	3.96 ± 0.40	2.87 ± 0.11	0.66 ± 0.04	0.96 ± 0.04
**2**	2.33 ± 0.04	4.57 ± 0.08	3.99 ± 0.21	0.91 ± 0.06	1.11 ± 0.06
**3**	3.58 ± 0.02	8.74 ± 0.23	3.71 ± 0.15	2.25 ± 0.08	2.68 ± 0.26
**4**	4.36 ± 0.19	6.87 ± 0.35	2.34 ± 0.14	2.14 ± 0.11	4.25 ± 0.11
[RuCl_2_(bipy)(dppp)]	40.93 ± 1.76	>50	26.06 ± 4.84	28.02 ± 0.81	35.59 ± 1.70
Bipy	>100	>100	>100	>100	>100
Dppp	>100	>100	>100	>100	>100
HFe	>100	>100	>100	>100	>100
HMFe	>100	>100	>100	>100	>100
HMTFe	>100	>100	>100	>100	>100
HMFFe	>100	>100	>100	>100	>100
cisplatin	29.1 ± 0.8	11.54 ± 1.19	12.43 ± 0.20	11.80 ± 0.80	37.02 ± 5.10

**Table 2:** Selectivity Inices (SI) for the MRC-5, A2780, and A2780cis Cell Lines^a^

	SI^1^	SI^2^	SI^3^	SI^4^
**1**	0.62	0.86	3.74	2.57
**2**	0.51	0.58	2.56	2.09
**3**	0.41	0.96	1.59	1.33
**4**	0.63	1.86	2.03	1.02
precursor	–	1.57	1.46	1.15
ligands	–	–	–	–
cisplatin	2.52	2.34	2.46	0.78

a SI^1^ = IC_50_(MRC-5)/IC_50_(A549). SI^2^ = IC_50_(MRC-5)/IC_50_(MDA-MB-231). SI^3^ = IC_50_(MRC-5)/IC_50_(A2780). SI^4^ = IC_50_(MRC-5)/IC_50_(A2780cis).

The results show a clear distinction in the biological activity between the free fenamic ligands and their corresponding metal complexes. At the maximum concentration tested (100 μM), the fenamic ligand alone showed no cytotoxicity. However, upon coordination to the ruthenium center, the resulting complexes (**1**–**4**) exhibited potent cytotoxic effects in all cell lines tested. These results emphasize the impact of metal coordination on the biological activities of the ligands.

The enhanced activity of complexes **1**–**4** compared to that of the precursor complex *cis*-[RuCl_2_(bipy)(dppp)] further underscores the role of fenamate ligands in modulating the biological behavior of the ruthenium center. Fenamate ligands not only stabilize the metal complex but also contribute bioactive functionalities. The higher cytotoxicity of these complexes compared to that of cisplatin, a commonly used chemotherapeutic agent, is noteworthy. The clinical application of cisplatin is frequently constrained by side effects and the development of resistance in various cancers. These complexes show potential in addressing resistance mechanisms and warrant further investigation. In particular, the IC_50_ values against cisplatin-resistant ovarian cancer cell line A2780cis ranged from 0.96 to 4.25 μM, underlining their potency. The relevant cytotoxic activity of ruthenium complexes with phosphine ligands is widely reported in the literature; many studies have shown that ruthenium complexes with dppp present IC_50_ values for the MDA-MB-231 cell line that ranged from 3 to 10 μM.^47,34,48,49^ These findings align with previous reports in which complexes **1** and **4** showed even lower IC_50_ values. Ruthenium complexes with ligands containing anti-inflammatory properties were reported elsewhere with gallic and salicylic acid, presenting compounds that were very active against the MDA-MB-231 cell line.^50,51^ Another study containing ruthenium complexes with mefenamic acid indicated IC_50_ values close to 145 μM for the A549 cell line,^52^ indicating that the compounds obtained in this report are promising (IC_50_ = 3.96–8.74 μM).

Thus, considering the promising results obtained against these two tumor lines with low IC_50_ values and good selectivity, complexes **1** and **2** were selected for detailed experiments using the A2780 cell line.

### Morphological and Clonogenic Assays

**Figure 3:**
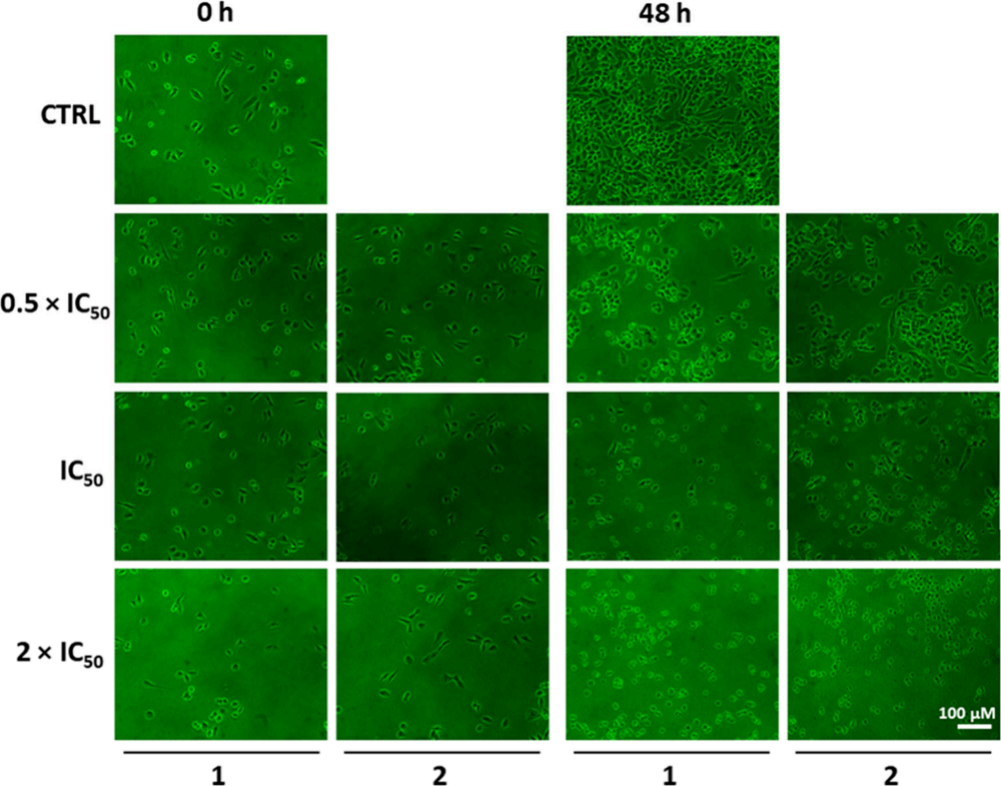
Representation of microscopic images illustrating the changes in the cellular morphology of A2780 ovarian cancer cells, 0 and 48 h after treatment with compounds **1** and **2** at concentrations of 0.5 × IC_50_, IC_50_, and 2 × IC_50_. The negative control was DMSO. The images were captured using a NIKON ECLIPSE TS100 microscope at 10× magnification.

**Figure 4:**
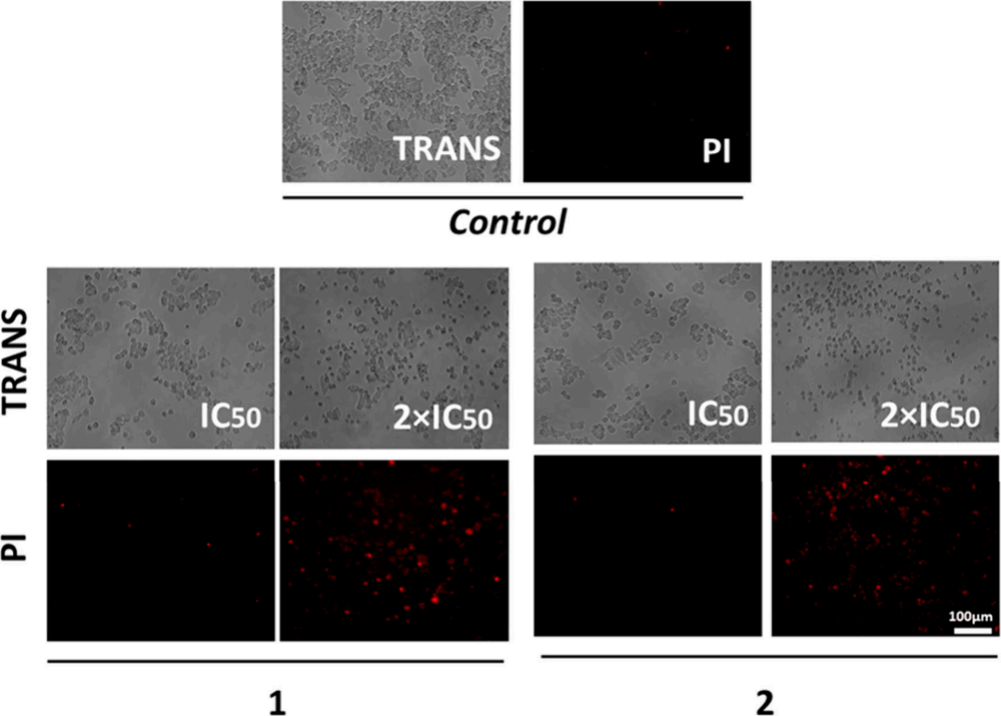
Illustration of fluorescence microscopic images showing changes in the morphology of the A2780 ovarian cancer cells. Propidium iodide fluorescence after treatment with compounds **1** and **2** for 48 h at concentrations of 0.5 × IC_50_, IC_50_, and 2 × IC_50_. The negative control used was DMSO. The images were captured using a CELENA microscope at 10× magnification.

**Figure 5:**
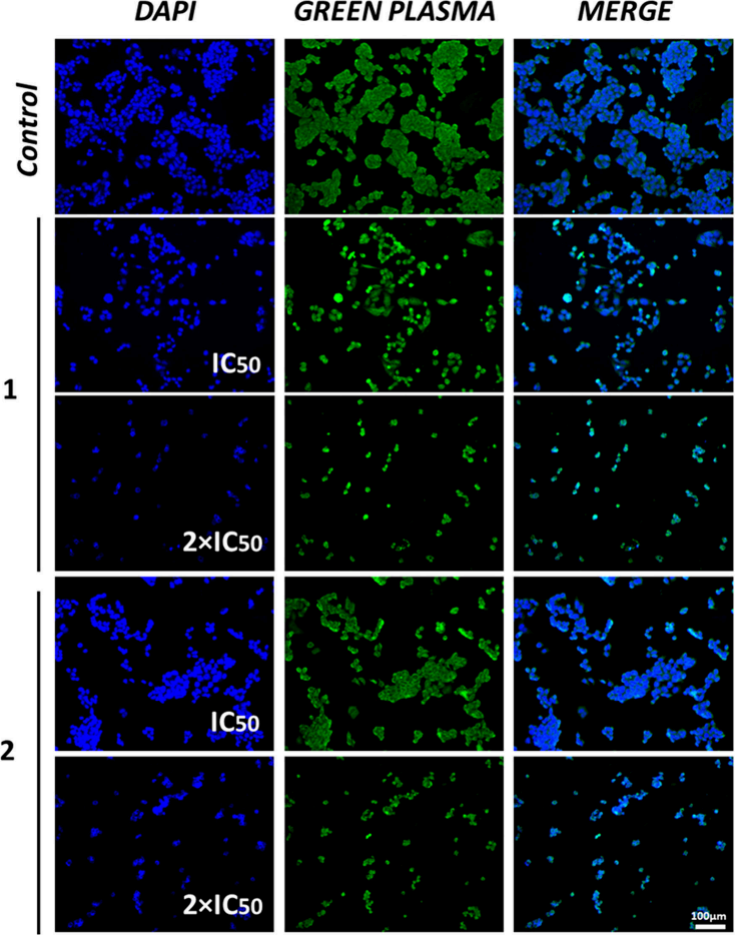
Representation of changes in the morphology of A2780 ovarian cancer cells by fluorescence microscopic images with Green Plasma and DAPI markers. Data were obtained after treatment with compounds **1** and **2** for 48 h at concentrations of 0.5 × IC_50_, IC_50_, and 2 × IC_50_. The negative control was DMSO. The images were captured using a CELENA microscope at 10× magnification.

**Figure 6:**
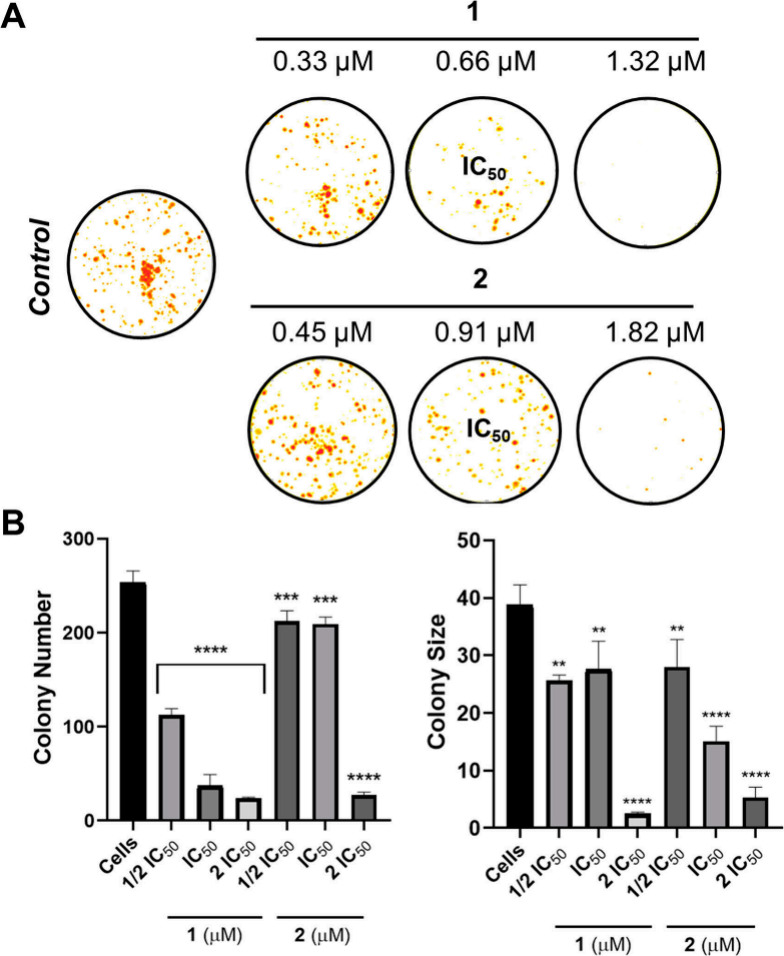
Effect of complexes **1** and **2** on colony formation of A2780 tumor cells (A) Images of colonies at different concentrations of the compounds. The experiment was conducted in triplicate. (B) Illustration of the quantitative data that represent the colony number and colony size related to the concentrations of the complexes. The negative control was DMSO, and the data are presented as the mean ± standard deviation of three independent measurements. The statistical analysis was conducted by using a one-way analysis of variance test (**p < 0.01, ***p < 0.001, and ****p < 0.0001).

Because compounds **1** and **2** showed promising selectivity results in A2780 cells, their effects on morphology and colony formation were further investigated. After treatment, in the presence and absence of **1** or **2**, the cells were monitored for ≤48 h. As presented in Figure 3, no significant changes are observed in the negative control. On the contrary, loss of adhesion and round cells are observed, mainly at higher concentrations, indicating cell damage. In a second experiment, the cells were stained with PI (propidium iodide), which was capable of penetrating damaged cells. Figure 4 demonstrates a significant increase in fluorescence intensity generated by PI as the concentration of the compounds increases, suggesting their ability to induce cellular damage and cell death.^53,54^ Also, the cells were treated with DAPI and Green Plasma. DAPI is commonly used for nuclear marking, primarily binding to adenine-thymine regions, while Green Plasma marks the plasma membrane.^55−57^ As shown in Figure 5, in addition to a reduction in cell density, the compounds caused a significant shortening of the plasma membrane, while the nucleus remained intact. This observation implies that the nucleus is not the primary target of the complexes, which is consistent with the findings from the DNA interaction experiments. Following these observations, a clonogenic assay was conducted to assess the impact of compounds **1** and **2** on colony formation. Treatment with compounds **1** and **2** at 0.5 × IC_50_ in A2780 cells significantly reduced both the number and size of colonies, demonstrating their ability to inhibit clonogenic survival in cancer cells (Figure 6).^58^

### Cellular Uptake by HR-CS GFAAS

Due to the higher cytotoxicity of complexes **1** and **2** against the A2780 cell line, experiments were performed to evaluate their cell internalization capacity. These studies are crucial to determine whether the complexes can efficiently penetrate the cell membrane and to correlate their antiproliferative effects with their uptake levels.^59^ A2780 cells were exposed to complexes **1** and **2** (1 μM) over a period of 4 h, and the cellular uptake of ruthenium was quantified using graphite furnace atomic absorption spectrometry.

**Figure 7:**
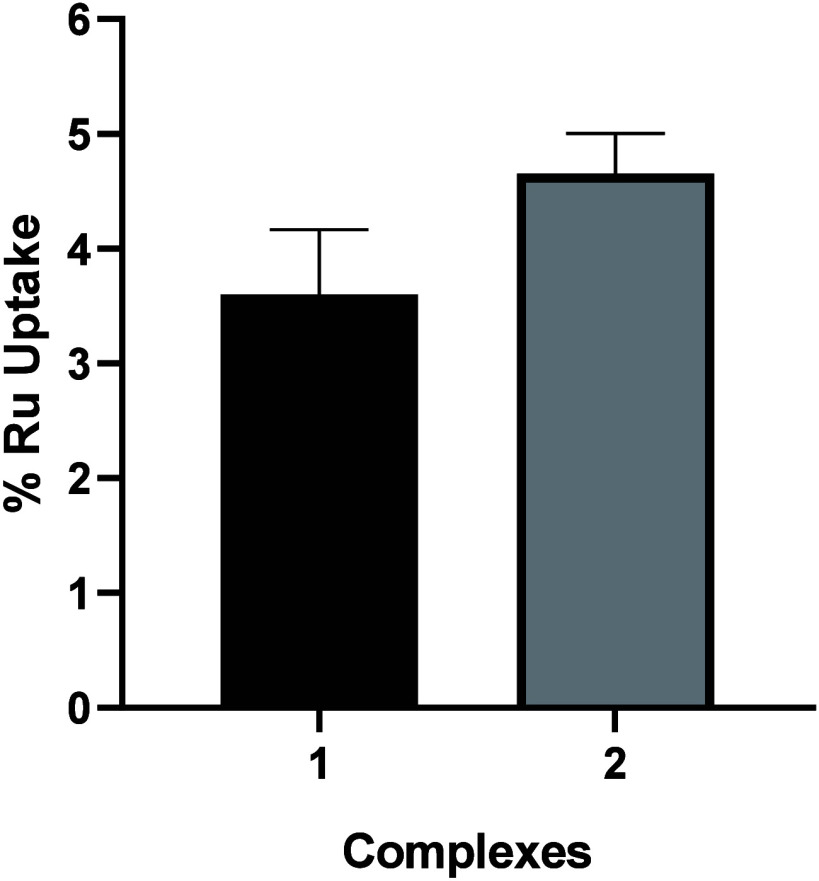
Percentage of ruthenium uptake in A2780 cells after incubation with complexes **1** and **2** at a concentration of 1 μM.

As shown in Figure 7 and detailed in Table S5, the amount of ruthenium internalized by A2780 cells after 4 h was 20.8 ± 3.2 ng (3.6%) for complex **1** and 26.0 ± 1.9 ng (4.5%) for complex **2**. These values are comparable, indicating that both complexes have a similar capacity for cellular internalization. This similarity is consistent with the close IC_50_ values observed for the two complexes, suggesting that their comparable levels of ruthenium uptake contribute significantly to their analogous cytotoxic profiles.

The efficient uptake of these complexes highlights their ability to cross the cell membrane, which is a critical factor in achieving their biological activity. The slight difference in internalization levels between complexes **1** and **2** could be attributed to differences in their structural or physicochemical properties, such as lipophilicity or charge distribution, which could influence their interaction with the cell membrane.

### 3D Tumor Spheroids

**Figure 8:**
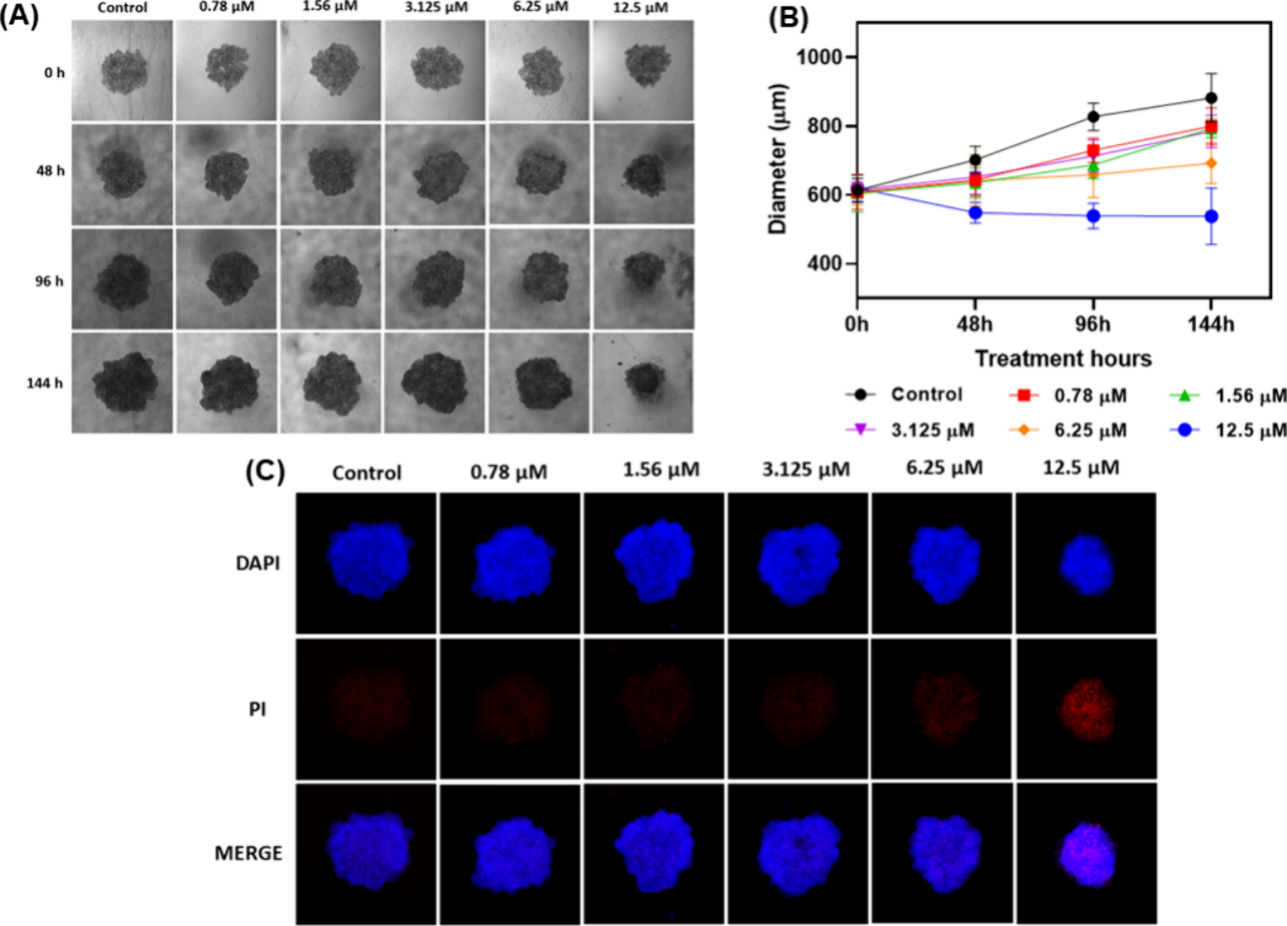
(A) Effect of complex **1**, at different concentrations, on the cell morphology of spheroids of the A2780 tumor line, for 144 h. (B) Graph of spheroid diameter vs treatment time. (C) Fluorescence microscopy with DAPI and PI markers, after treatment for 144 h. The images were recorded using a CELENA microscope at 4x magnification. The negative control was DMSO.

Considering the cytotoxicity results from *in vitro* 2D monolayer assays, complex **1** was selected to be investigated in 3D tumor spheroids (Figure 8), given that these models exhibit distinct proliferation rates and drug sensitivities compared to those of 2D assays. In this experiment, the spheroids were monitored for 144 h after treatment with **1** at several concentrations (0.78–12.5 μM). As shown in panels A and B of Figure 7, spheroid growth, assessed by measuring the diameter, was significantly compromised only at a concentration of 12.5 μM. As observed, at this concentration there is a decrease in the diameter, followed by stabilization, after treatment for 48 h. Notably, this concentration is ∼20 times higher than the IC_50_ value determined from the 2D assays, underscoring the enhanced resistance of the cells cultured in a 3D model. These findings underscore the necessity to refine *in vitro* methodologies to better elucidate the potential of novel compounds. Such improvements are crucial for increasing the likelihood of identifying critical insights early in the drug discovery and development process, thereby minimizing errors in subsequent stages. After treatment for 144 h, double staining with both DAPI and PI was performed. The images indicated that increasing concentrations of the compound led to a corresponding increase in the fluorescence intensity of the PI marker, suggesting an increase in the rate of cell death.

## Conclusion

Four new ruthenium(II) complexes, using bipy and dppp as ancillary ligands in combination with fenamic acid derivatives [fenamate (**1**), mefenamate (**2**), tolfenamate (**3**), and flufenamate (**4**)], were successfully synthesized and characterized. These complexes exhibited weak interactions with the DNA biomolecule. Nevertheless, all of the complexes showed good cytotoxic activity against the tumor cell lines tested [A549 (lung), MDA-MB-231 (breast), A2780 (ovarian), and A2780cis (ovarian cisplatin-resistant)], with activities that were higher than those of the free ligands and the precursor complex, which confirm the effectiveness of the strategy adopted, consisting of coordinating bioactive molecules with ruthenium, in which the complexes formed are more active than the free molecules. In particular, the activities of the complexes were better than that of the reference drug cisplatin, particularly against ovarian tumor cell lines (A2780 and A2780cis). All of the complexes showed low IC_50_ values against the A2780cis tumor line, suggesting their potential to overcome cisplatin resistance, a critical challenge in the development of new metallodrugs. The ability of these complexes to avoid common resistance mechanisms highlights their promise as candidates for the treatment of resistant tumors. Complexes **1** and **2** also demonstrated the ability to alter cell morphology, reduce cell density, promote cell death, damage the plasma membrane, and inhibit colony formation, underscoring their potent biological effects. Complex **1** exhibited growth-inhibitory effects on 3D spheroids, demonstrating its potential for anticancer activity in a more physiologically relevant model and suggesting promise for future *in vivo* applications. Overall, these findings emphasize the promising therapeutic potential of this complex class, particularly in overcoming the limitations of existing chemotherapeutic agents, such as cisplatin.

## Experimental Section

### Reagents and Materials

All solvents were obtained from Synth (P.A.) or Merck (P.A.) without further purification. The reagents, including ruthenium trichloride hydrate (RuCl_3_·*n*H_2_O), triphenylphosphine (PPh_3_), 2,2′-bipyridine (bipy), 1,3-bis(diphenylphosphino)propane (dppp), fenamic acid (HFen), mefenamic acid (HMFen), tolfenamic acid (HTFen), flufenamic acid (HFFen), and potassium hexafluorophosphate (KPF_6_), were purchased from Sigma-Aldrich.

### Physical Measurements

The molar conductivity of complexes **1**–**4** was measured using a Meter Laboratory conductivity meter (model CDM2300). The compound solutions were dissolved in DMSO, resulting in a concentration of 1 × 10^–3^ mol L^–1^. Melting point measurements were taken in MARCONI NA 301 equipment at a rate of 5 °C min^–1^. The infrared (FT-IR) spectra were recorded using a Bomem-Michelson FT spectrophotometer (model MB-102) and KBr (potassium bromide) pellets. Elemental analyses were carried out using a CHNS Element Analyzer (Thermo Scientific, Fisons EA-1108 model). The ultraviolet and visible absorption spectra were acquired using a Hewlett-Packard (HP) 8452A diode-array spectrophotometer using cuvettes with an optical path of 1 cm containing solutions of the complexes of known concentrations, in DMSO. Mass spectrometry measurements were conducted in positive mode using an Agilent 6545 ESI-QTOF-MS instrument (electrospray ionization quadrupole time-of-flight, Agilent Technologies, Santa Clara, CA). Full-scan mass spectrometry data were acquired across a mass range of 150–1500 Da. Samples were prepared in acetonitrile (MeCN) and subjected to FIA at a flow rate of 0.3 mL min^–1^, with an injection volume of 3.0 μL, at a temperature of 38 °C. The mobile phase was composed of H_2_O with 0.1% formic acid and MeCN in a 5:95 ratio, with an analysis time of 3.0 min. The ESI source parameters were configured with the following settings: capillary voltage of 2100 V, nozzle voltage of 300 V, gas temperature of 320 °C, drying gas flow rate of 12 L min^–1^, nebulizer pressure of 35 psi, sheath gas temperature of 300 °C with a flow rate of 10 L min^–1^, fragmentor voltage of 110 V, and skimmer voltage of 65 V. The data were processed and interpreted using Qualitative Navigator version B.08.000. The NMR analyses were carried out on a BRUKER DRX 400 MHz spectrometer belonging to the UFSCar Chemistry Department. The spectra of the complexes were obtained in DMSO and chloroform.

Single crystals of complex **1** were obtained by recrystallization of a methanolic solution. The X-ray data were collected on an XtaLAB Synergy Dualflex HyPix diffractometer at room temperature. The crystal structure of complex **1** was determined using the ShelXT 2018/2^60^ structure solution program with the Intrinsic Phasing method, in which Olex2^61^ was employed as the graphical interface. The crystal structure of complex **1** was refined by least-squares minimization, using a 2019/2 version of ShelXL^62^

### Synthesis

The syntheses of complexes **1**–**4** were carried out in an argon atmosphere. The [RuCl_2_(PPh_3_)_3_]^63^ and *cis,trans*-[RuCl_2_(PPh_3_)_2_(bipy)]^64^ complexes used as precursors were synthesized according to the literature. For the synthesis of the precursor complex *cis-*[RuCl_2_(bipy)(dppp)], 0.560 g of *cis,trans*-[RuCl_2_(PPh_3_)_2_(bipy)] (0.65 mmol) and 0.325 g of dppp (0.78 mmol) were added to 40 mL of deaerated toluene under argon. The reaction system was refluxed and heated for 72 h, and the precipitate was vacuum filtered and washed with ethyl ether. The yield was 79%.

The new complexes [Ru(L)(bipy)(dppp)]PF_6_ (**1–4**) were synthesized in 10 mL of methanol and 10 mL of dichloromethane previously deaerated in an argon atmosphere by adding 0.16 mmol of ligand L (where L = fenamic acid, mefenamic acid, tolfenamic acid, and flufenamic acid, respectively) with two drops of Et_3_N, to form the fenamate ion. After agitation for 30 min, 0.14 mmol of the precursor complex *cis*-[RuCl_2_(bipy)(dppp)] and 0.16 mmol of KPF_6_ were added. After 24 h, the volume was reduced and the complex was precipitated with distilled water. The product was filtered, washed with water and ethyl ether, and dried under vacuum.

#### [Ru(Fen)(bipy)(dppp)]PF_6_ (**1**)

Yield: 75%. Elemental analysis (%) for C_50_H_44_N_3_O_2_F_6_P_3_Ru: experimental (theoretical) C, 58.09 (58.48); H, 4.24 (4.32); N, 4.45 (4.09). Molar conductance (Ω^–1^ cm^2^ mol^–1^, DMSO): 20.51. Melting point (°C): 163. UV–vis (DMSO, 1.0 × 10^–3^ M): λ (nm) [ε (M^–1^ cm^–1^)] 299 (36 267), 368 (13 046), 425 (6584). Infrared (cm^–1^): 3325 (νNH), 3056 (νCH), 1586 (ν_as_COO^–^), 1497 (νC=C), 1446 (ν_as_COO^–^), 843 (νP–F), 556 (δP–F), 522 (νRu–P), 467 (νRu–O). *m*/*z* [M]^+^: experimental (theoretical) 882.1956 (882.19467). ^1^H NMR (400 MHz, DMSO-*d*_6_, 298 K): δ 8.92 (s, 1H of the N–H group), 8.72–6.04 [m, an overlap of aromatic protons of phenyl groups of dppp (20H), bipy (8H), and fenamic acid (9H)], 3.28–2.01 (m, 6H, aliphatic protons of dppp). ^13^C{^1^H} NMR (100 MHz, DMSO-*d*_6_, 298 K): δ 182.97 (C–O), 160–155 (C–N), 148–113 (aromatic carbon of phenyl groups of dppp, bipy, and fenamic acid), 28–21 (aliphatic carbon of dppp). ^31^P{^1^H} NMR (162 MHz, DMSO-*d*_6_, 298 K): δ (d, 51.55; 32.31; ^2^*J* = 45.0 Hz).

#### [Ru(MFen)(bipy)(dppp)]PF_6_ (**2**)

Yield: 83%. Elemental analysis (%) for C_52_H_48_N_3_O_2_F_6_P_3_Ru: experimental (theoretical) C, 59.36 (59.20); H, 4.87 (4.59); N, 3.92 (3.98). Molar conductance (Ω^–1^ cm^2^ mol^–1^, DMSO): 21.28. Melting point (°C): 165. UV–vis (DMSO, 1.0 × 10^–3^ M): λ (nm) [ε (M^–1^ cm^–1^)] 297 (26 801), 361 (11 186), 421 (8205). Infrared (cm^–1^): 3323 (νNH), 3050 (νCH), 1578 (ν_as_COO^–^), 1491 (νC=C), 1439 (ν_as_COO^–^), 841 (νP–F), 557 (δP–F), 510 (νRu–P), 434 (νRu–O). *m*/*z* [M]^+^: experimental (theoretical) 910.2267 (910.22597). ^1^H NMR (400 MHz, DMSO-*d*_6_, 298 K): δ 8.64 (s, 1H of the N–H group), 8.68–6.04 [m, an overlap of aromatic protons of phenyl groups of dppp (20H), bipy(8H), and mefenamic acid (7H)], 3.31–1.99 (m, 6H, aliphatic protons of dppp), 2.24 (s, 3H of C–H_3_ of mefenamic acid), 1.81 (s, 3H of C–H_3_ of mefenamic acid). ^13^C{^1^H} NMR (100 MHz, DMSO-*d*_6_, 298 K): δ 183.39 (C–O), 160–155 (C–N), 148–113 (aromatic carbon of phenyl groups of dppp, bipy, and mefenamic acid), 28–14 (aliphatic carbon of dppp and C–H_3_ of mefenamic acid). ^31^P{^1^H} NMR (162 MHz, DMSO-*d*_6_, 298 K): δ (d, 51.89; 32.35; ^2^*J* = 45.4 Hz).

#### [Ru(TFen)(bipy)(dppp)]PF_6_ (**3**)

Yield: 72%. Elemental analysis (%) for C_51_H_45_N_3_O_2_ and F_6_P_3_Ru: experimental (theoretical) C, 56.11 (56.96); H, 4.52 (4.22); N, 3.37 (3.91). Molar conductance (Ω^–1^ cm^2^ mol^–1^, DMSO): 20.11. Melting point (°C): 167. UV–vis (DMSO, 1.0 × 10^–3^ M): λ (nm) [ε (M^–1^ cm^–1^)] 297 (32 006), 355 (13 286), 421 (4416). Infrared (cm^–1^): 3308 (νNH), 3058 (νCH), 1578 (ν_as_COO^–^), 1498 (νC=C), 1437 (ν_as_COO^–^), 841 (νP–F), 560 (δP–F), 511 (νRu–P), 430 (νRu–O). *m*/*z* [M]^+^: experimental (theoretical) 930.1725 (930.17135). ^1^H NMR (400 MHz, DMSO-*d*_6_, 298 K): δ 8.86 (s, 1H of the N–H group), 8.68–6.21 [m, an overlap of aromatic protons of phenyl groups of dppp (20H), bipy(8H), and tolfenamic acid (7H)], 3.41–2.19 (m, 6H, aliphatic protons of dppp), 2.11 (s, 3H of C–H_3_ of tolfenamic acid). ^13^C{^1^H} NMR (100 MHz, DMSO-*d*_6_, 298 K): δ 185.26 (C–O), 162–157 (C–N), 150–116 (aromatic carbon of phenyl groups of dppp, bipy, and tolfenamic acid), 30–17 (aliphatic carbon of dppp and C–H_3_ of tolfenamic acid). ^31^P{^1^H} NMR (162 MHz, DMSO-*d*_6_, 298 K): δ (d, 54.05; 34.60; ^2^*J* = 45.2 Hz).

#### [Ru(FFen)(bipy)(dppp)]PF_6_ (**4**)

Yield: 79%. Elemental analysis (%) for C_51_H_43_N_3_O_2_F_9_P_3_Ru: experimental (theoretical) C, 55.42 (55.95); H, 3.24 (3.96); N, 4.08 (3.84). Molar conductance (Ω^–1^ cm^2^ mol^–1^, DMSO): 20.54. Melting point (°C): 160. UV–vis (DMSO, 1.0 × 10^–3^ M): λ (nm) [ε (M^–1^ cm^–1^)] 297 (33 371), 357 (12 046), 422 (4511). Infrared (cm^–1^): 3308 (νNH), 3056 (νCH), 1583 (ν_as_COO^–^), 1438 (νC=C), 1448 (ν_as_COO^–^), 1288 (νCF_3_), 837 (νP–F), 555 (δP–F), 506 (νRu–P), 428 (νRu–O). *m*/*z* [M]^+^: experimental (theoretical) 950.1831 (950.18206). ^1^H NMR (400 MHz, CDCl_3_-*d*_6_, 298 K): δ 9.02 (s, 1H of the N–H group), 8.64–6.09 [m, an overlap of aromatic protons of phenyl groups of dppp (20H), bipy (8H), and flufenamic acid (8H)], 3.25–2.35 (m, 6H, aliphatic protons of dppp). ^13^C{^1^H} NMR (100 MHz, CDCl_3_-*d*_6_, 298 K): δ 183.25 (C–O), 159–155 (C–N), 148–113 (aromatic carbon of phenyl groups of dppp, bipy, and flufenamic acid), 31–21 (aliphatic carbon of dppp). ^31^P{^1^H} NMR (162 MHz, DMSO/D_2_O, 298 K): δ (d, 51.80; 32.70; ^2^*J* = 44.9 Hz).

### DNA Binding Experiments

#### Viscosity

The viscosity of DNA with complexes **1**–**4** was determined using an Ostwald viscosimeter at 25 °C. All samples (4.0 mL) were prepared in Tris-HCl buffer (pH 7.4), containing 30% DMSO. The concentration of CT-DNA was kept constant at 100 μM, while the concentration of the complexes ranged from 5 to 40 μM, leading to [complex]:[CT-DNA] molar ratios of 0.05–0.40. The flow times were registered with a digital stopwatch in five replicates. All specific viscosity values [(η/η_0_)^1/3^] were plotted versus the [complex]:[CT-DNA] molar ratio, in which η and η_0_ correspond to the relative viscosity of CT-DNA in the presence and absence, respectively, of complexes **1**–**4**. By using the expression (*t* – *t*_DNA_)/*t*_DNA_, where *t* is the observed flow time and *t*_DNA_ is the flow time of DNA, we calculated the values of η and η_0_.

#### Fluorescence Spectroscopy

To a solution of CT-DNA (125 μM) and Hoechst 33258 (2.5 μM) at different molar ratios were added complexes **1**–**4**, in DMSO. Solutions of CT-DNA-Hoechst in Tris-HCl buffer (4.5 mM Tris-HCl, 0.5 mM Tris base, and 50 mM of NaCl), with 10% DMSO, were prepared. Subsequently, 200 μL of each solution was added to a 96-well opaque plate and analyzed in a Sinergy H1-Biotek fluorimeter in the range of 370–670 nm, with excitation at 343 nm, at 37 °C.

#### Circular Dichroism

The CD titration experiments were performed with a JASCO J-815 spectropolarimeter at room temperature (25 °C). Solutions of CT-DNA [100 μM, Tris-HCl buffer (pH 7.4)] with several [complex]:[CT-DNA] molar ratios (0.10–0.40) were incubated for 24 h at 37 °C. The spectra were recorded from 240 to 500 nm, using a quartz cuvette with an optical path length of 0.5 cm. The scanning rate was 200 nm min^–1^.

### Biological Studies

#### Cell Culture and Cytotoxicity Assay

In this study, A549 (lung), MDA-MB-231 (breast), A2780 (ovarian), and A2780cis (ovarian cisplatin-resistant) cancer cells and MRC-5 (lung) normal cells were used. DMEM (Dulbecco’s modified Eagle’s medium) was supplemented with 10% fetal bovine serum (FBS), penicillin (100 units mL^–1^), gentamicin (50 mg L^–1^), and amphotericin (25 μg mL^–1^) and used to cultivate the A549, MDA-MB-231, and MRC-5 cells. A2780 and A2780cis cells were cultivated in RPMI 1640 medium (Roswell Park Memorial Institute) with 10% fetal bovine serum (FBS), penicillin (100 units mL^–1^), gentamicin (50 mg L^–1^), and amphotericin (25 μgmL^–1^). The cell lines were grown in culture bottles and incubated at a CO_2_ concentration of 5% and 37 °C.

Cell viability was investigated via the MTT [3-(4,5-dimethylthiazol-2-yl)-2,5-diphenyltetrazolium bromide] assay. This method consists of the capacity of enzymes present in living cells, especially in mitochondria, to reduce MTT to formazan, a purple-colored compound. This process is directly proportional to mitochondrial activity and, consequently, cell viability.^65^ For the assays, 1.5 × 10^4^ cells/well were seeded in 96-well flat-bottom cell culture plates (KASVI). The plates were incubated for 24 h; then, different concentrations of the complexes were pipetted, and the plates were treated for an additional 48 h. To complete the treatment, 50 μL of MTT (1 mg/mL in PBS) was added to each well, followed by incubation for 3 h. The formazan crystals that formed were solubilized in isopropanol, and the absorbance was read with a microplate reader (BioTek, Epoch) at 540 nm. Cell viability (IC_50_) was assessed with GraphPad Prism 8.

#### Morphological Assay

For morphological analysis, A2780 cancer cells (0.3 × 10^5^ cells/well) were added to a 12-well plate in RPMI medium. After 24 h, different concentrations of complexes **1** and **2** were introduced into the cells. Possible morphological changes were evaluated at 0 and 48 h using an inverted optical microscope (NIKON ECLIPSE TS100) coupled with a Motcam 1SP camera, with a 10× objective lens.

Additionally, 1.5 × 10^4^ A2780 cells/well were plated in 96-well plates in RPMI medium for the fluorescence morphology experiments. After treatment for 48 h, 100 μL (1 μg mL^–1^) of the fluorescent marker PI (propidium iodate) was added. For the morphological assessment using the fluorescent markers DAPI (4′,6-diamidino-2-phenylindole dilactate) and Green Plasma (CellMask), the cells were fixed in methanol and 80 μL (1 μg mL^–1^) portions of the markers were added. Images were taken using a CELENA microscope (Logos Biosystems).

#### Clonogenic Survival Assay

The A2780 cancer cells were used for the clonogenic assay, in which a total of 400 cells/well were seeded in a six-well plate in RPMI medium, and after incubation for 24 h, different concentrations of complexes **1** and **2** were added. The plates containing the complexes were incubated (37 °C and 5% of CO_2_) for 48 h. The culture medium was replaced with fresh medium, and the system was incubated again for 7 days under the same condition. Thereafter, all culture media were removed from the plate, and the cells were washed with PBS and fixed with a 3:1 methanol/acetic acid mixture. Then the cells were colored with a 0.5% crystal violet solution in water for 30 min. Images were taken, and the colonies counted using ImageJ. The experiment was also carried out in triplicate.

#### Cellular Uptake by HR-CS GFAAS

A2780 cancer cells (2 × 10^6^ cells/plate) were seeded in Petri dishes. After 24 h, complexes **1** and **2** (1 μM) were added to the cells for 4 h. Subsequently, the RPMI medium was removed, and the cells were washed with phosphate-buffered saline (PBS), trypsinized, and counted. The ruthenium absorbance measurements were conducted with an Analytik Jena ContrAA 700 graphite furnace atomic absorption spectrometer (Jena, Germany), equipped with solid sampling tubes. The absorbance measurements were performed at 349.8945 nm, with the peak volume selected absorbance (PVSA) corresponding to three pixels. The heating program for the graphite furnace, used for the quantitative determination of ruthenium, is depicted in Table S4.^66^ The analytical working solution of 50 mg L^–1^ Ru was prepared by diluting standard solutions of 1000 mg L^–1^ Ru (Specsol, São Paulo, Brazil) and acidifying with 0.5% (v/v) HNO_3_. To establish the calibration curve, appropriate aliquots of the analytical working solution were added to the atomizer to achieve concentrations of 0.1, 0.3, 0.6, 0.9, 1.2, and 1.5 ng of ruthenium. The cell suspension containing the internalized complexes was diluted with 500 μL of deionized water, acidified with 0.5% (v/v) HNO_3_, and homogenized using a vortex mixer (IKA model V1). For the determination of Ru by SS HR-CS GFAAS, 10 μL aliquots of the samples were transferred to the platform and introduced automatically into the graphite furnace, in which all measurements were carried out in triplicate.

#### 3D Tumor Spheroids

In a 96-well Bioprint Kit [Magnetic 3D Cell Culture Technology (m3D)], the 3D culture experiment was carried out. Initially, to the A2780 cells was added 150 μL of a solution containing nanoparticles (NanoShuttle - PL). The A2780 cells were added to a 96-well repellent culture plate after 24 h. The resulting 1500 cells/well were placed under magnetic drives to form spheroids through the magnetic forces of the magnets. Furthermore, the plate was stored in an incubator at a CO_2_ concentration of 5% and 37 °C. The formation of spheroids and their growth were monitored by a Digital Imaging System microscope (CELENA S, Logos Biosystems). Once the spheroids were formed, complex **1** at different concentrations (0.78, 1.56, 3.12, 6.25, and 12.5 μM) was added, and the effect of the compound on the spheroids was monitored for 144 h. Finally, 100 μL (1 μg mL^–1^) portions of the fluorescent markers DAPI and PI were added.

## Supporting Information Available

Figures and tables providing infrared, UV–vis, HRESI-MS, and ^31^P{^1^H}, ^1^H, and ^13^C{^1^H} NMR spectra, stability studies, DNA interactions, morphologic assays, and cellular uptake data for the complexes (PDF)
