# Supplementary material for: Ru(II)-Fenamic-Based Complexes as Promising Human Ovarian Antitumor Agents: DNA Interaction, Cellular Uptake, and Three-Dimensional Spheroid Models
Source: Inorg Chem. 2025 Feb 18;64(8):3707–18. doi: 10.1021/acs.inorgchem.4c04344 (PMC11884657; doi:10.1021/acs.inorgchem.4c04344)
Supplement: Supplementary file 1 — ic4c04344_si_001.pdf [file ic4c04344_si_001.pdf]

Supporting Information

Ru(II)-Fenamic Based Complexes as Promising Human Ovarian Antitumor Agents: DNA Interaction, Cellular Uptake, and 3D Spheroid Models

Tamara Teixeira[a,b]*, Marcos V. Palmeira-Mello[b], Pedro Henrique Machado[a,b], Carlos A. F. Moraes[b], Camila Pinto[c], Rayane Costa[d], Wladimir Badaró[d], José Gomes Neto [d], Javier Ellena[c], Fillipe Vieira Rocha[b], Alzir A. Batista[b], Rodrigo S. Correa[a*]

[a] Department of Chemistry, Institute of Exact and Biological Sciences, Federal University of Ouro Preto (UFOP), Zip code 35402-136, Ouro Preto, Minas Gerais, Brazil.

[b] Department of Chemistry, Federal University of São Carlos (UFSCar), Zip code 13565-905, São Carlos, São Paulo, Brazil.

[c] Institute of Physics of São Carlos, University of São Paulo (IFSC/USP), Zip code 13566-590, São Carlos, São Paulo, Brazil.

[d] Institute of Chemistry, São Paulo State University (UNESP), Zip code 14800-900, Araraquara, São Paulo, Brazil.

* Corresponding authors:

Tamara Teixeira (e-mail: tamara.teixeira.296@gmail.com) and Rodrigo S. Corrêa (e-mail: rodrigocorrea@ufop.edu.br)

IR data

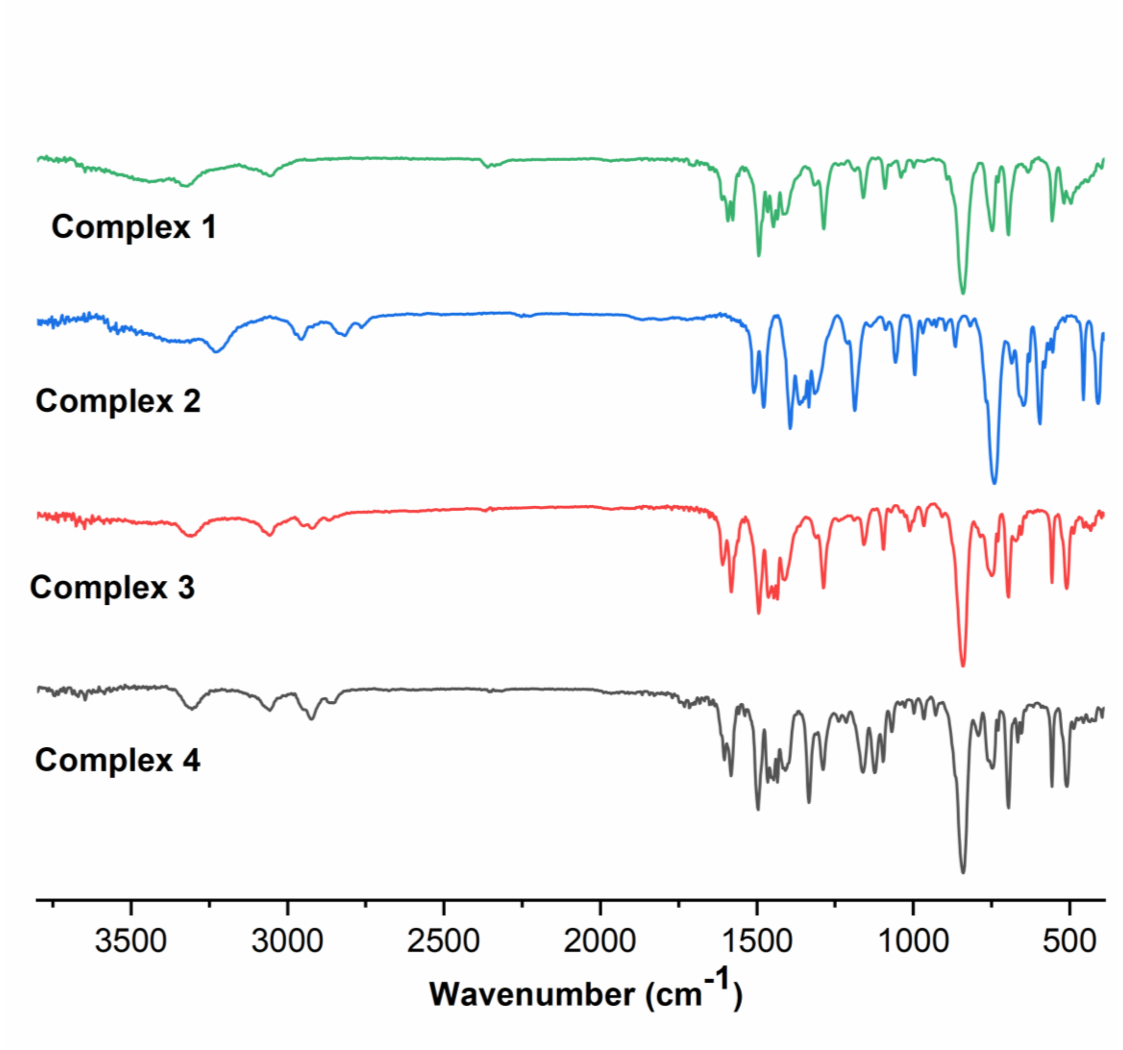

Figure S1 - Absorption spectra in the infrared region of the complexes [Ru(Fen)(bipy)(dppp)]PF_6 (Complex **1**), [Ru(MFen)(bipy)(dppp)]PF_6 (Complex **2**), [Ru(TFen)(bipy)(dppp)]PF_6 (Complex **3**) and [Ru(FFen)(bipy)(dppp)]PF_6 (Complex **4**), in KBr.

UV/vis spectra

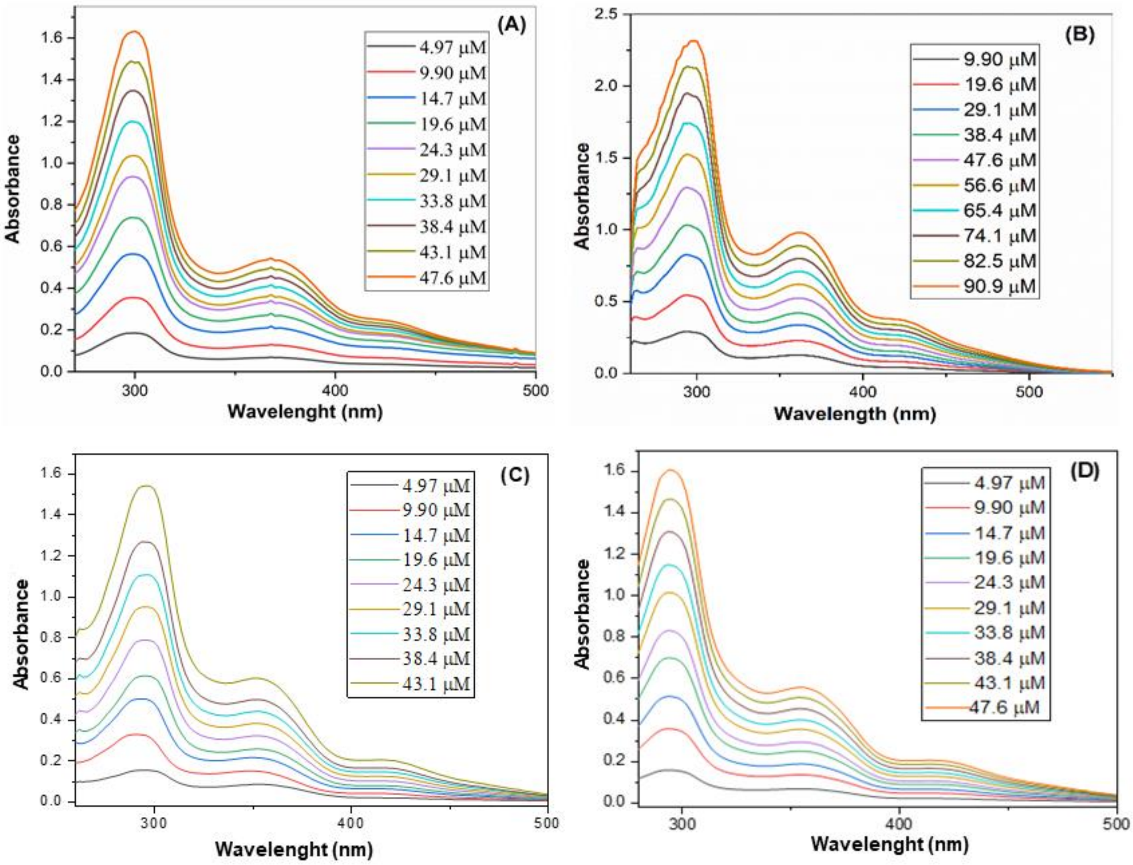

Figure S2 - Electronic absorption spectra in the UV-Vis region for complexes (A) 1, (B) 2, (C) 3 and (D) 4, in DMSO, in different concentrations.

Mass spectrometry

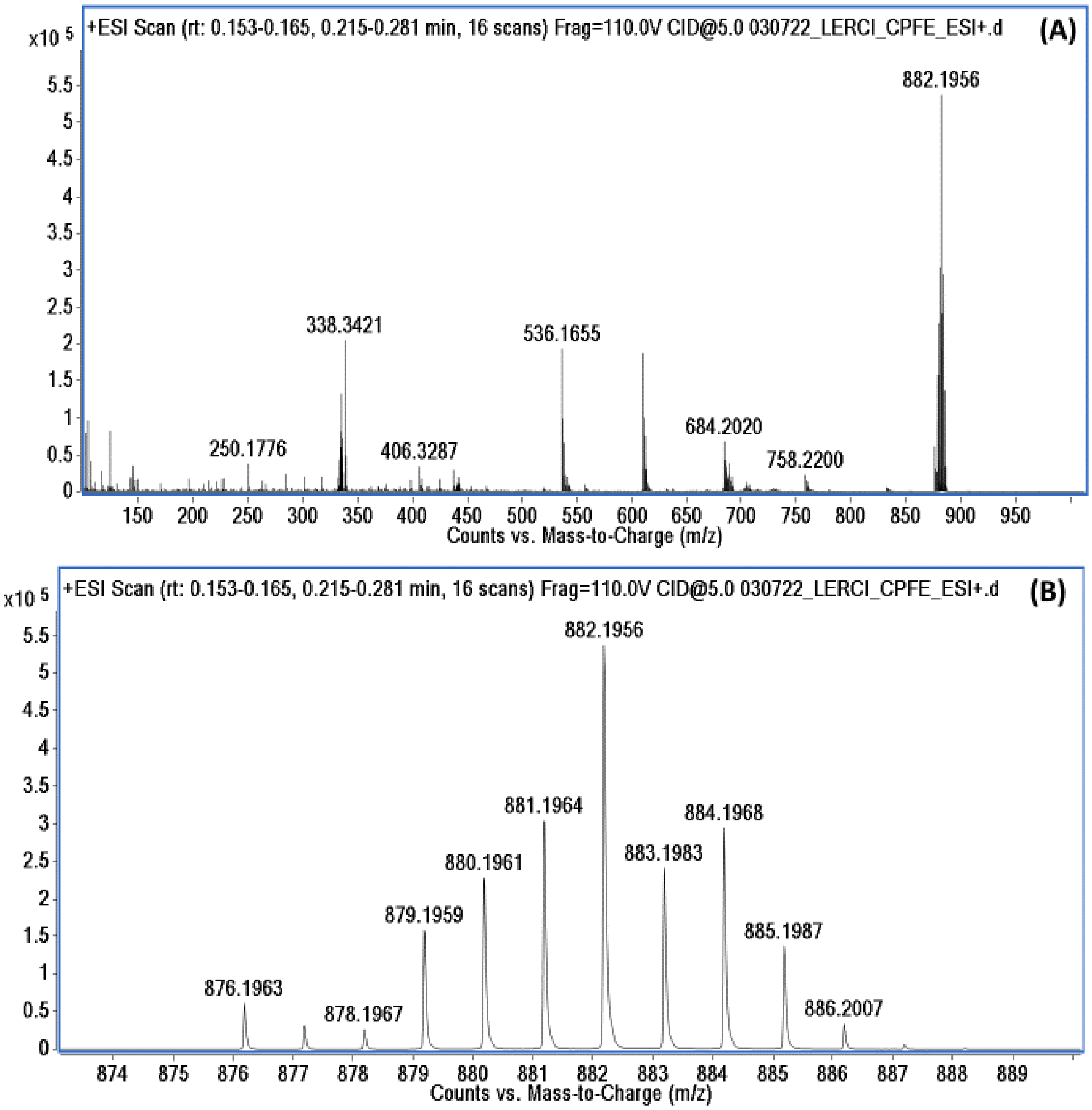

Figure S3 - HRESI-MS spectra of complex 1. A: HRESI-MS full scan mode of Complex 1, *m/z* 882.1956 $[M]^+$ (calcd. for $C_{50}H_{44}N_3O_2P_2Ru^+$). B: Isotopic pattern of *m/z* 882.1956.

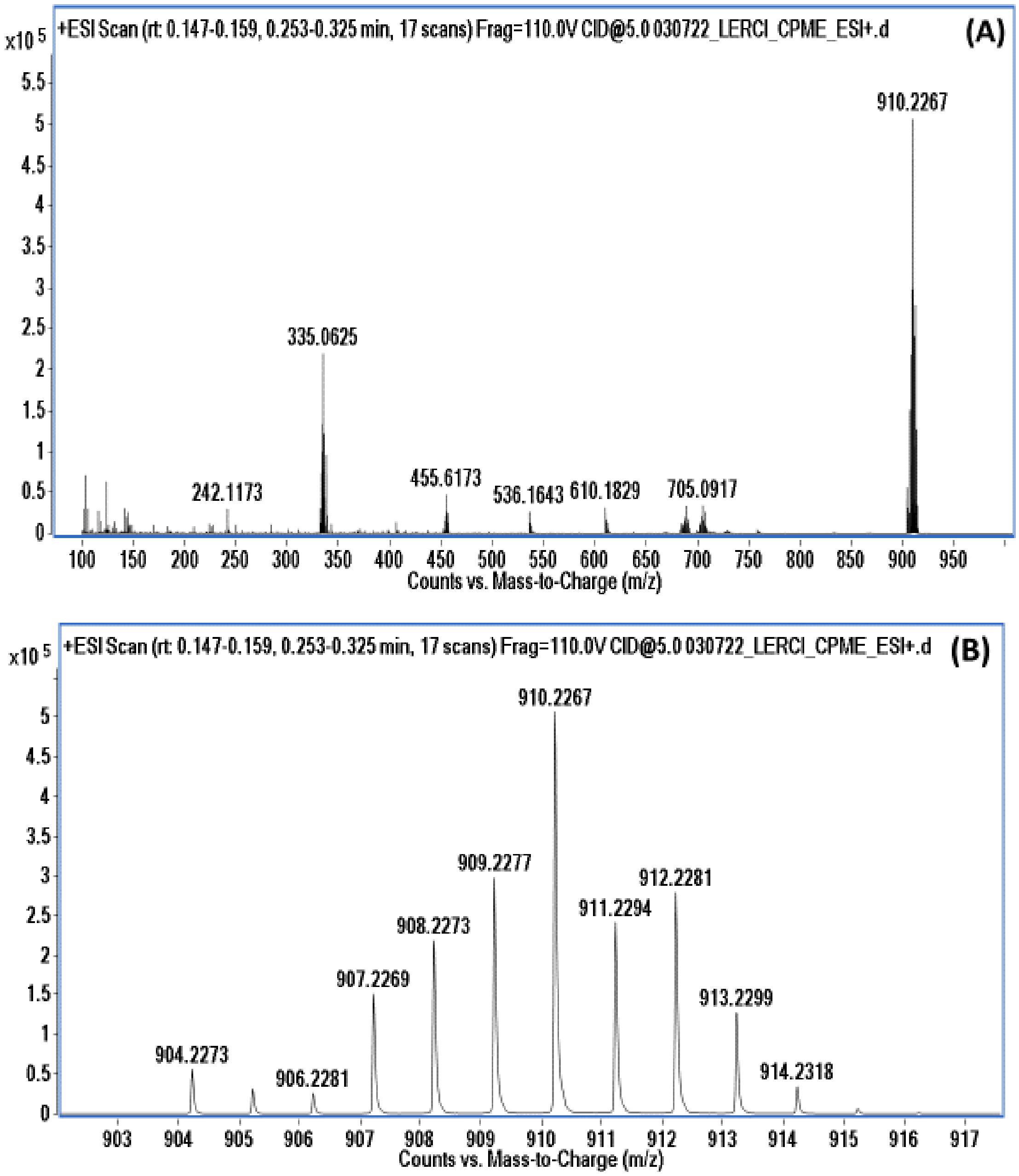

Figure S4 - HRESI-MS spectra of complex 2. A: HRESI-MS full scan mode of Complex 2, *m/z* 910.2267 $[M]^+$ (calcd. for $C_{52}H_{48}N_3O_2P_2Ru^+$). B: Isotopic pattern of *m/z* 910.2267.

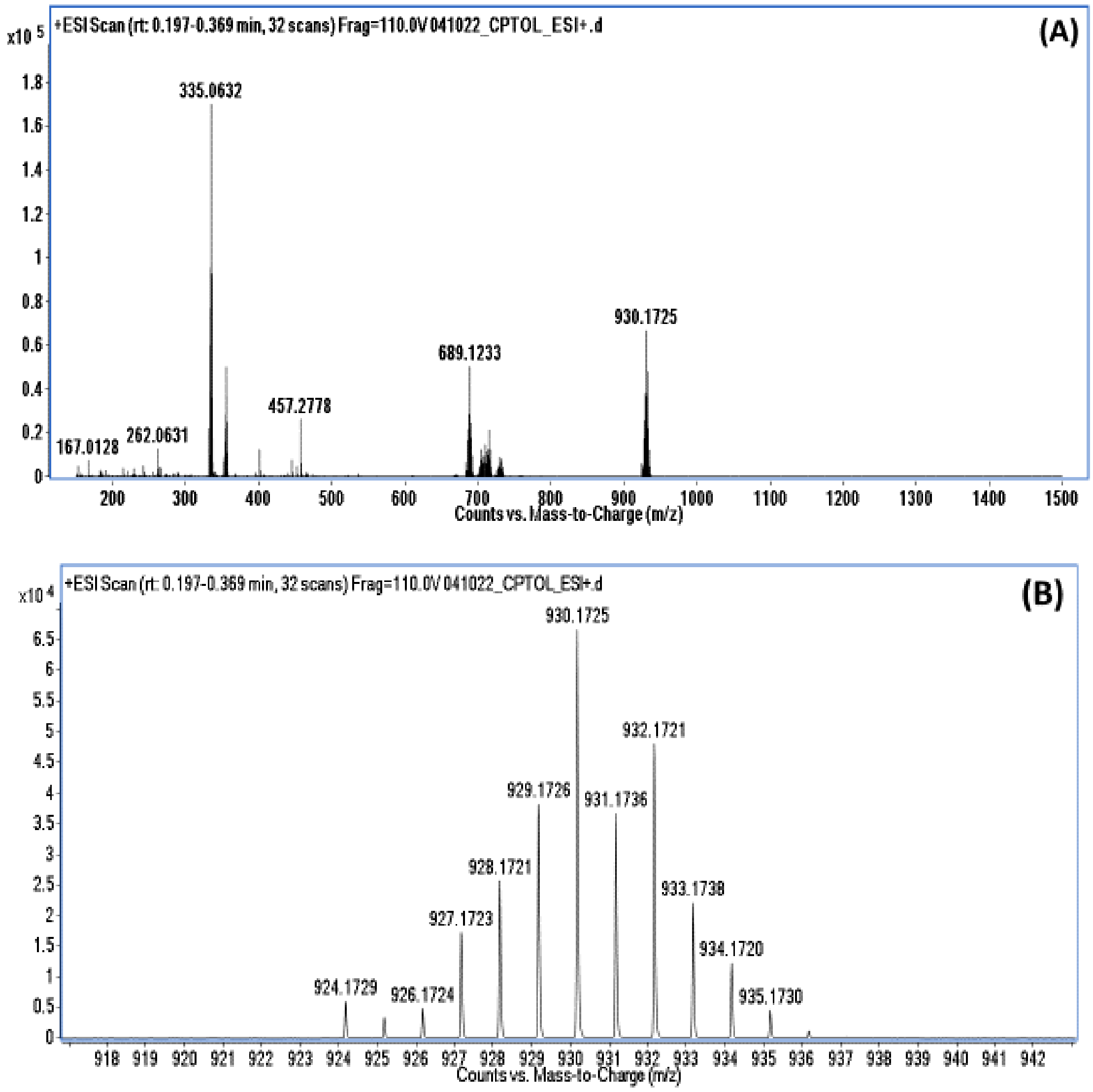

Figure S5 - HRESI-MS spectra of complex 3. A: HRESI-MS full scan mode of Complex 3, *m/z* 930.1725 $[M]^+$ (calcd. for $C_{51}H_{45}ClN_3O_2P_2Ru^+$). B: Isotopic pattern of *m/z* 930.1725.

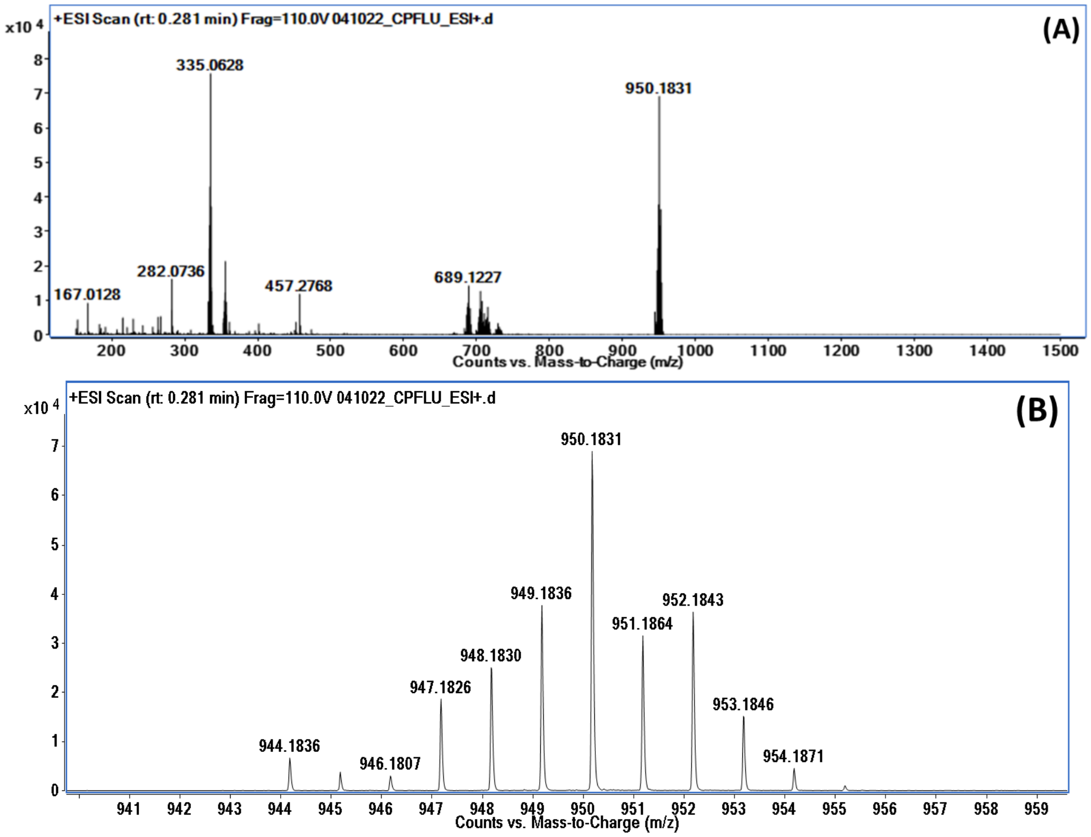

Figure S6 - HRESI-MS spectra of complex 4. A: HRESI-MS full scan mode of Complex 4, *m/z* 950.1831 $[M]^+$ (calcd. for $C_{51}H_{43}F_3N_3O_2P_2Ru^+$). B: Isotopic pattern of *m/z* 950.1831.

Table S1 - Theoretical and experimental data obtained by mass spectrometry.

Complex	Theoretical	Experimental	Error (ppm)
**1**	882.19467	882.1956	1.05 ppm
**2**	910.22597	910.2267	0.8 ppm
**3**	930.17135	930.1725	1.23 ppm
**4**	950.18206	950.1831	1.09 ppm

NMR spectra

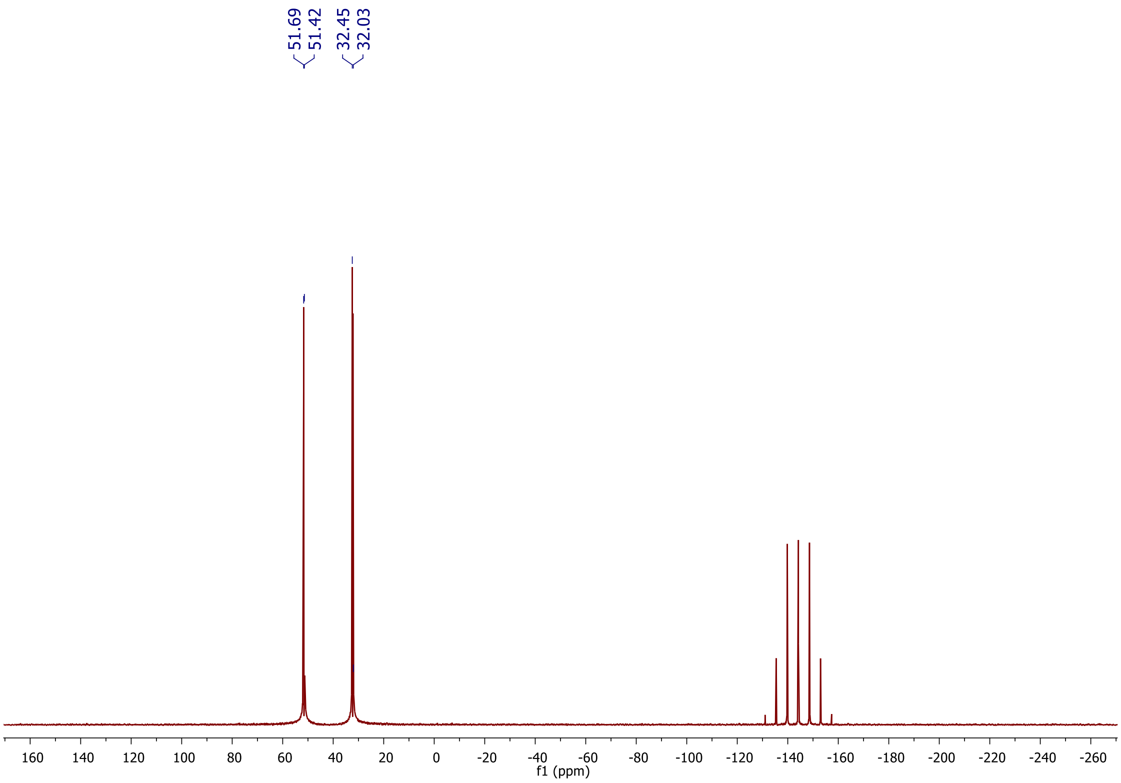

Figure S7 – $^{31}P\{^{1}H\}$ NMR spectrum of $[Ru(Fen)(bipy)(dppp)]PF_6$ (1) in DMSO-d_6 at 298K.

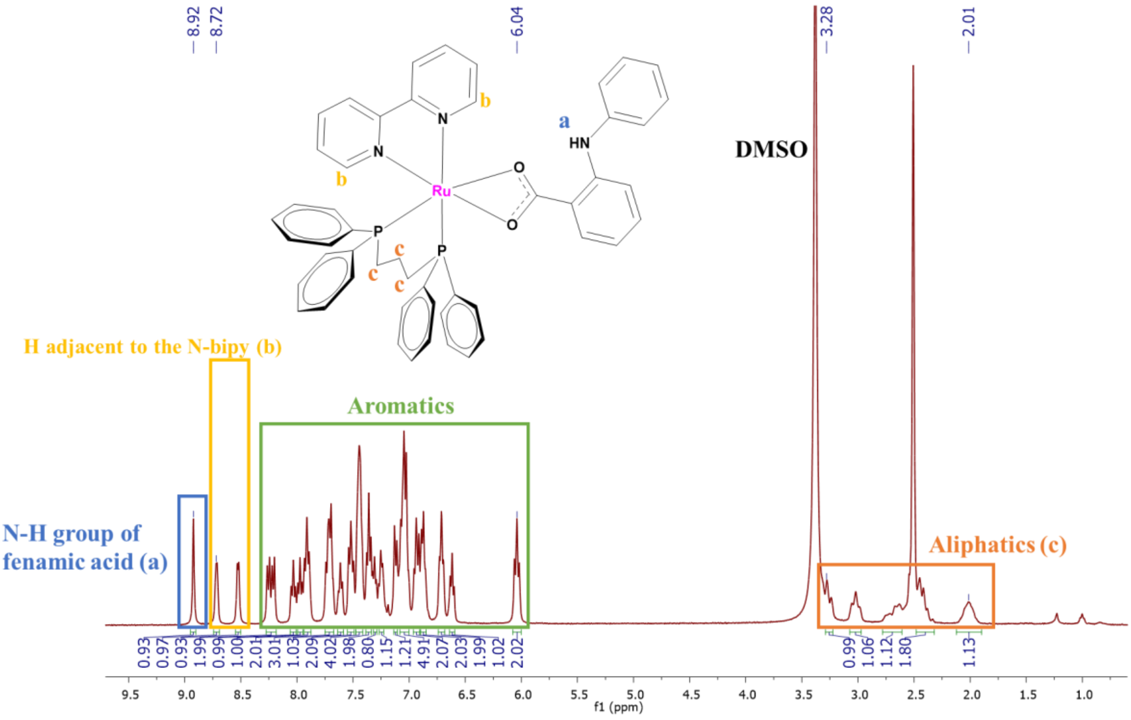

Figure S8 - ^{1}H NMR spectrum of $[Ru(Fen)(bipy)(dppp)]PF_6$ (1) in DMSO-d_6 at 298K.

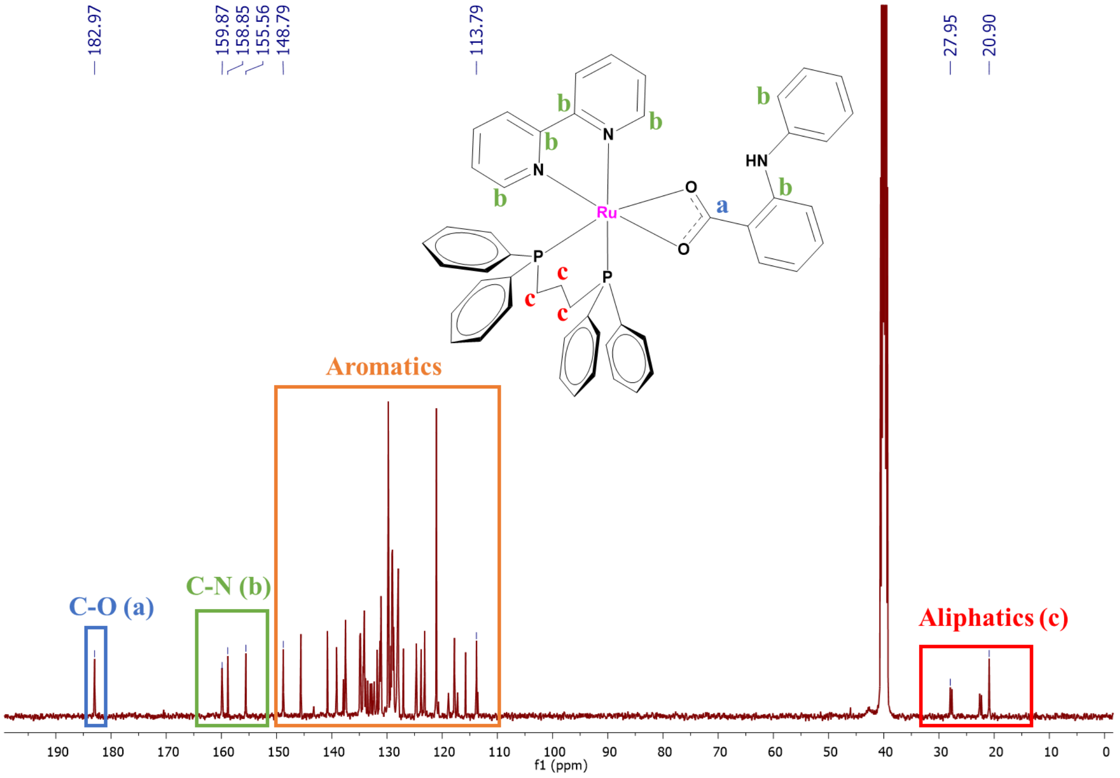

Figure S9 – ^{13}C NMR spectrum of [Ru(Fen)(bipy)(dppp)]PF_6 (1) in DMSO-d_6 at 298K.

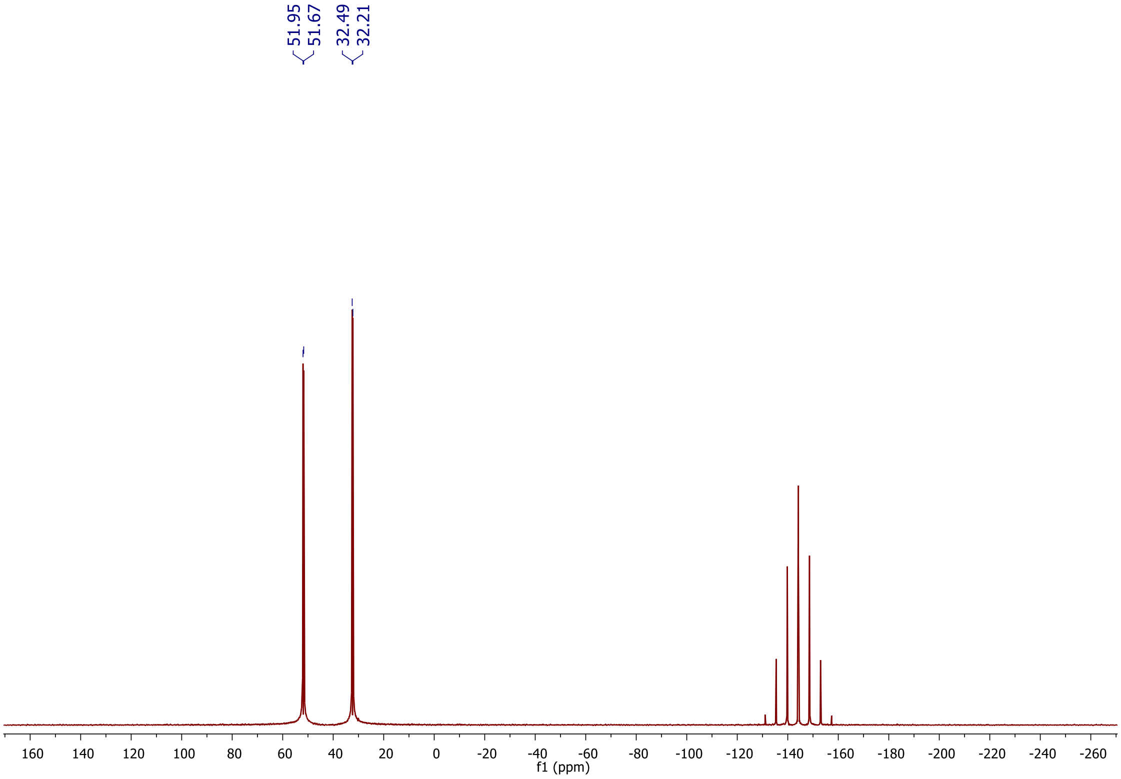

Figure S10 – $^{31}P\{^1H\}$ NMR spectrum of [Ru(MFen)(bipy)(dppp)]PF_6 (2) in DMSO-d_6 at 298K.

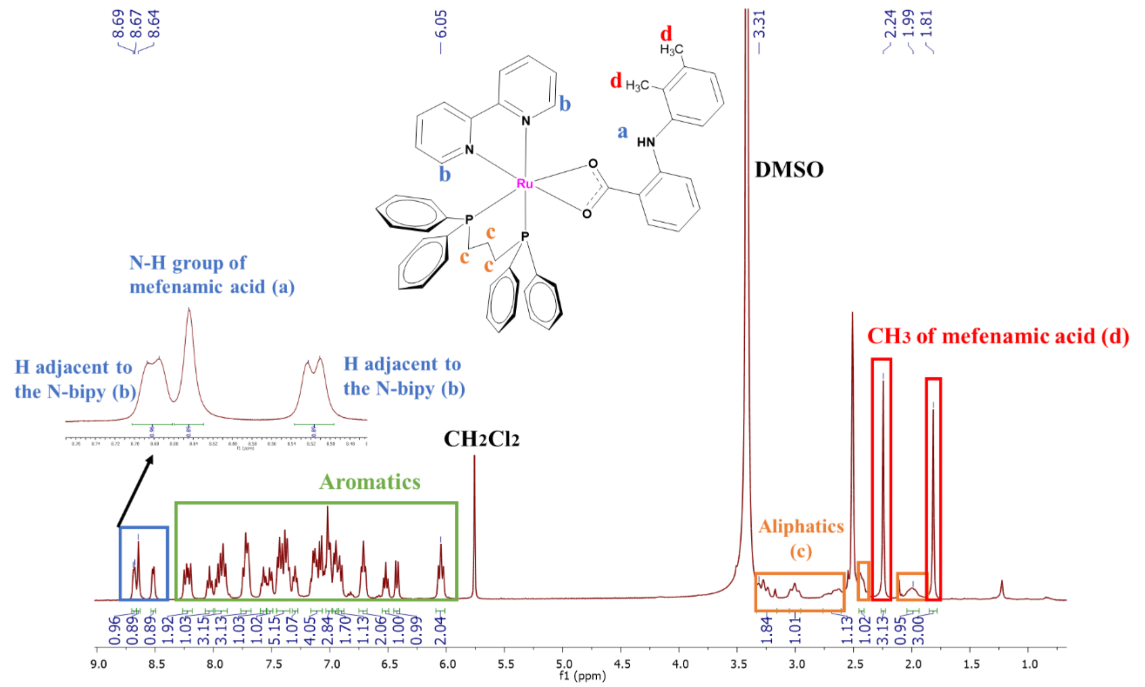

Figure S11 - ^{1}H NMR spectrum of [Ru(MFen)(bipy)(dppp)]PF_6 (2) in DMSO-d_6 at 298K.

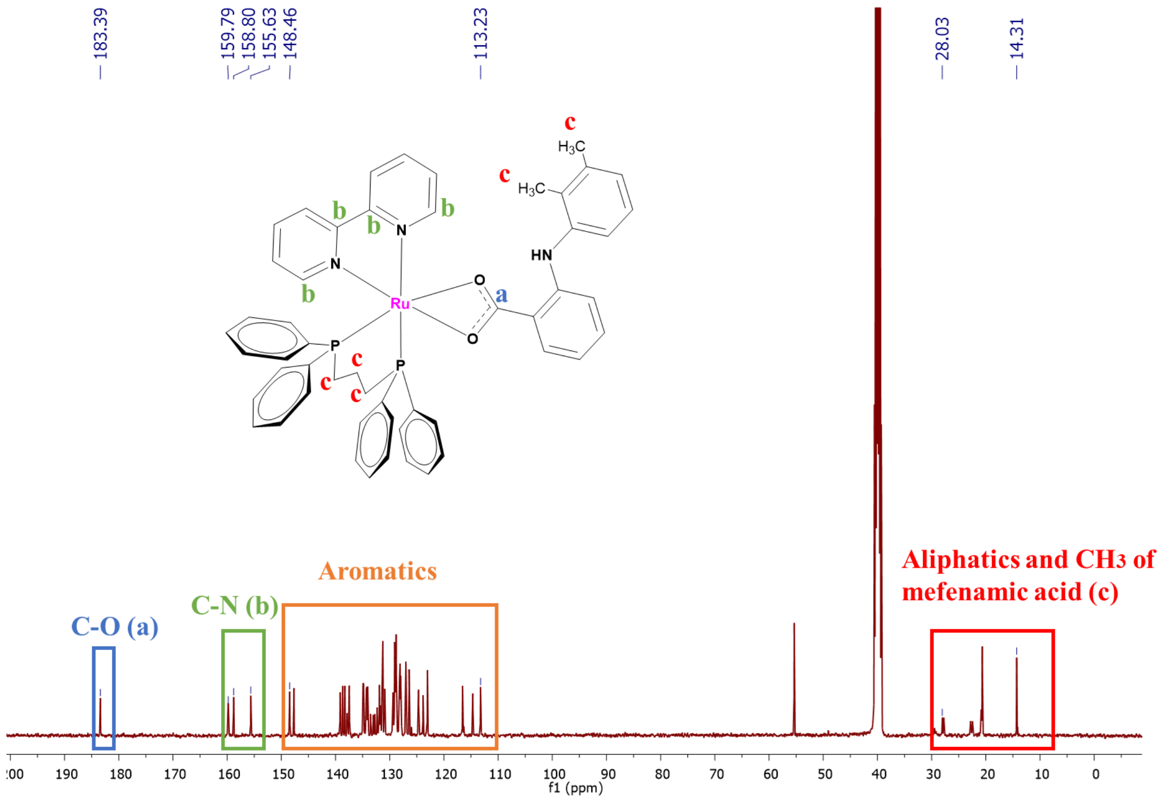

Figure S12 – ^{13}C NMR spectrum of [Ru(MFen)(bipy)(dppp)]PF_6 (2) in DMSO-d_6 at 298K.

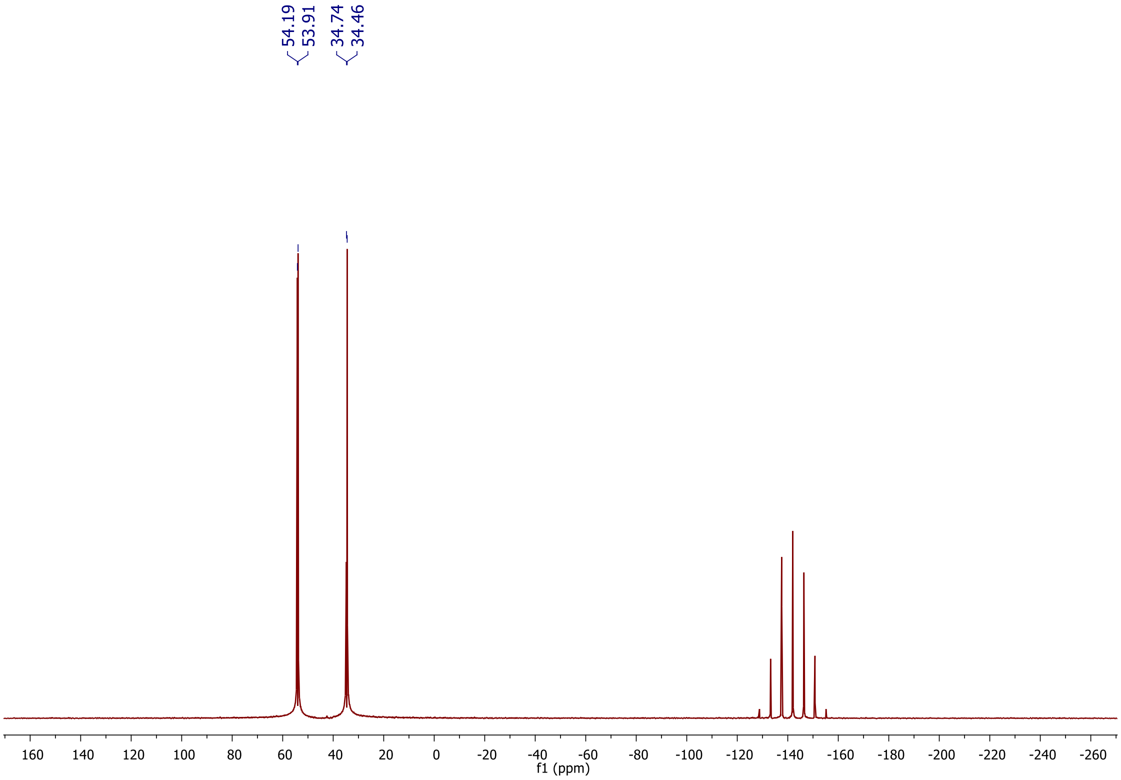

Figure S13– $^{31}P\{^{1}H\}$ NMR spectrum of $[Ru(TFen)(bipy)(dppp)]PF_6$ (3) in DMSO-d_6 at 298K.

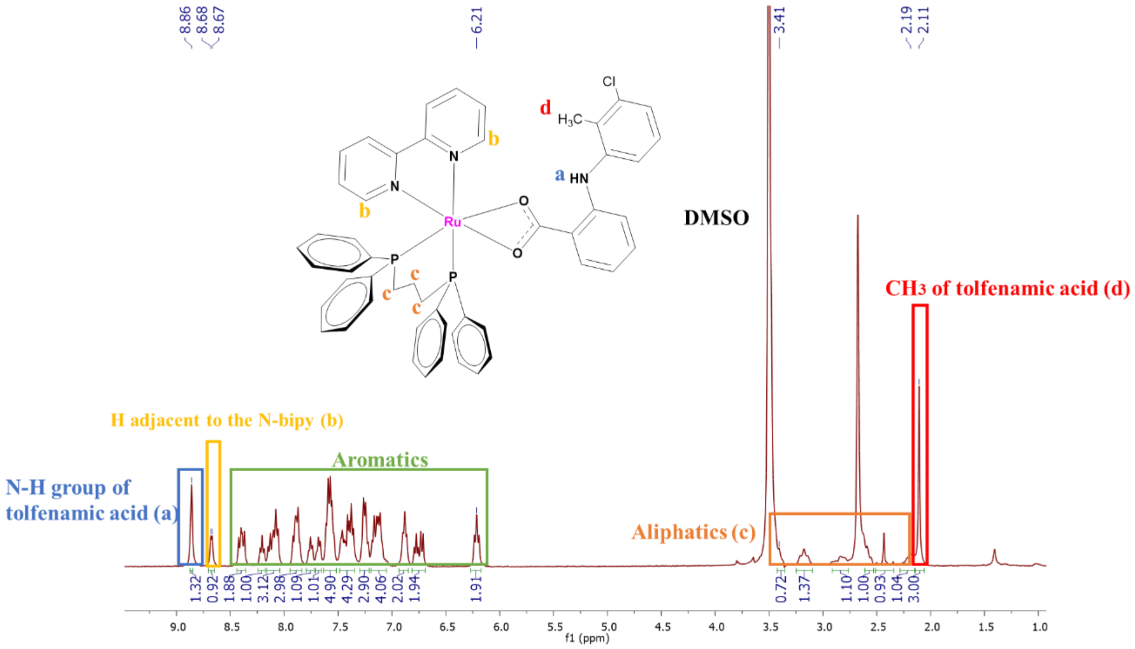

Figure S14 - ^{1}H NMR spectrum of $[Ru(TFen)(bipy)(dppp)]PF_6$ (3) in DMSO-d_6 at 298K.

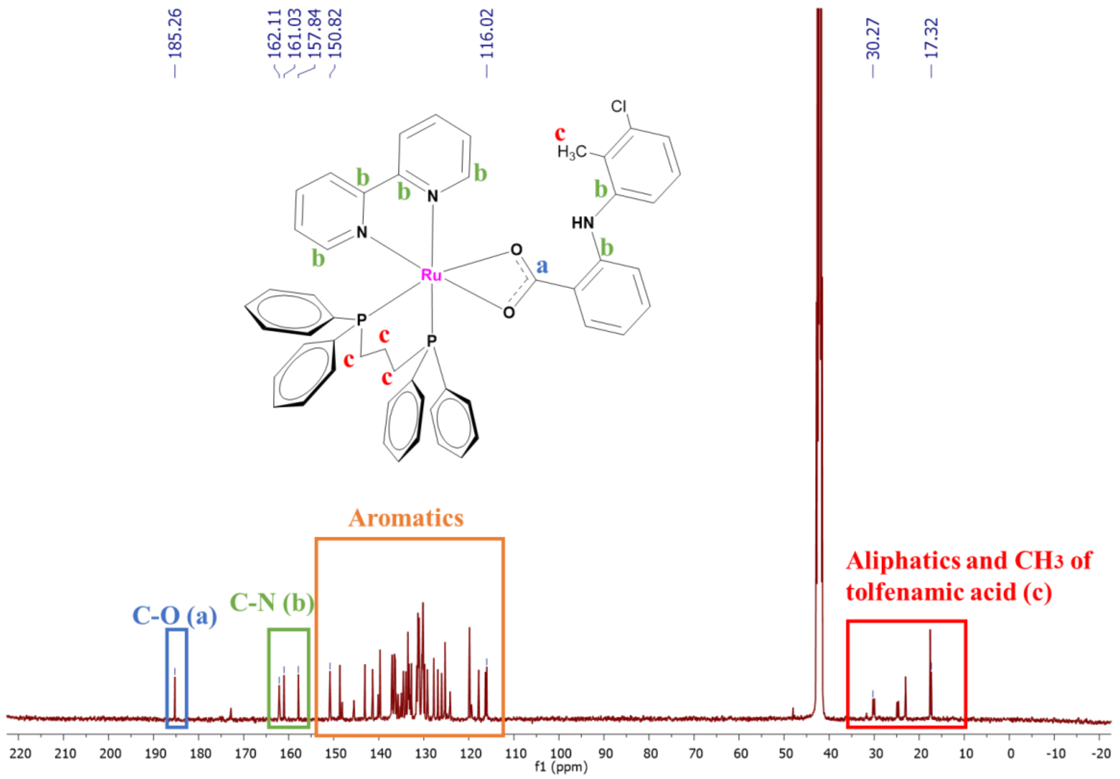

Figure S15 – ^{13}C NMR spectrum of [Ru(TFen)(bipy)(dppp)]PF_6 (3) in DMSO-d_6 at 298K.

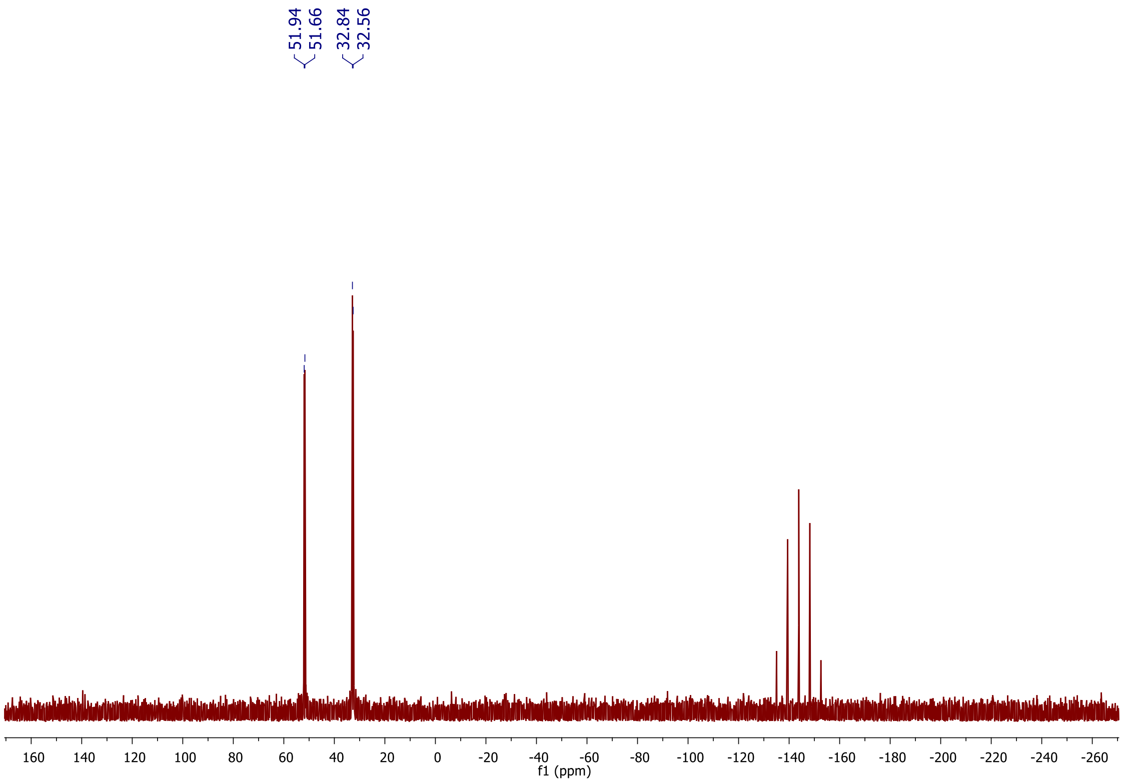

Figure S16 – $^{31}P\{^1H\}$ NMR spectrum of [Ru(FFen)(bipy)(dppp)]PF_6 (4) in DMSO/D_2O at 298K.

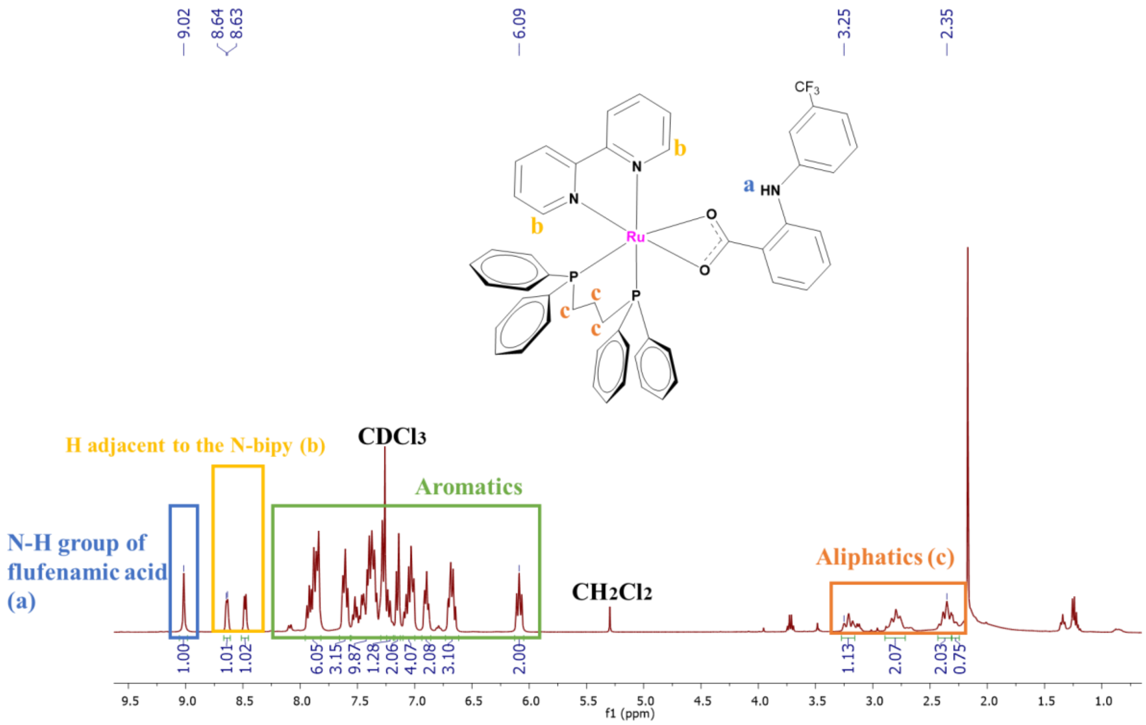

Figure S17 - ^{1}H NMR spectrum of $[Ru(FFen)(bipy)(dppp)]PF_6$ (4) in $CDCl_3$-d_6 at 298K.

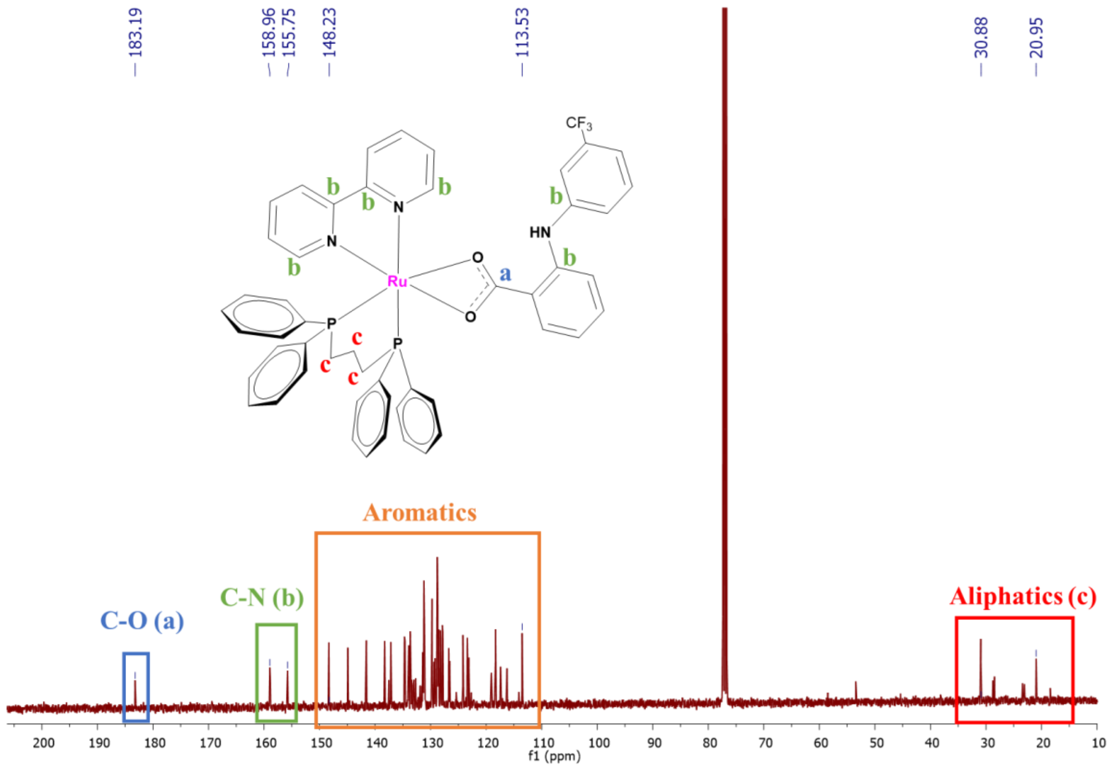

Figure S18 – ^{13}C NMR spectrum of $[Ru(FFen)(bipy)(dppp)]PF_6$ (4) in $CDCl_3$-d_6 at 298K.

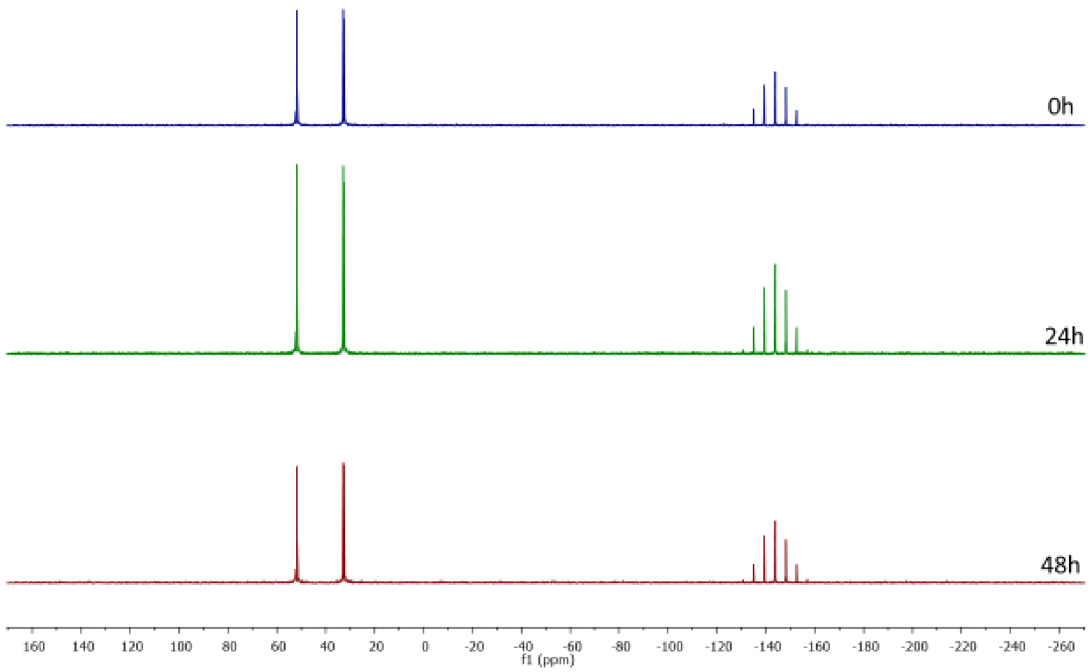

Figure S19 – $^{31}P\{^{1}H\}$ spectra of complex 1, in DMSO/D_2O at different times.

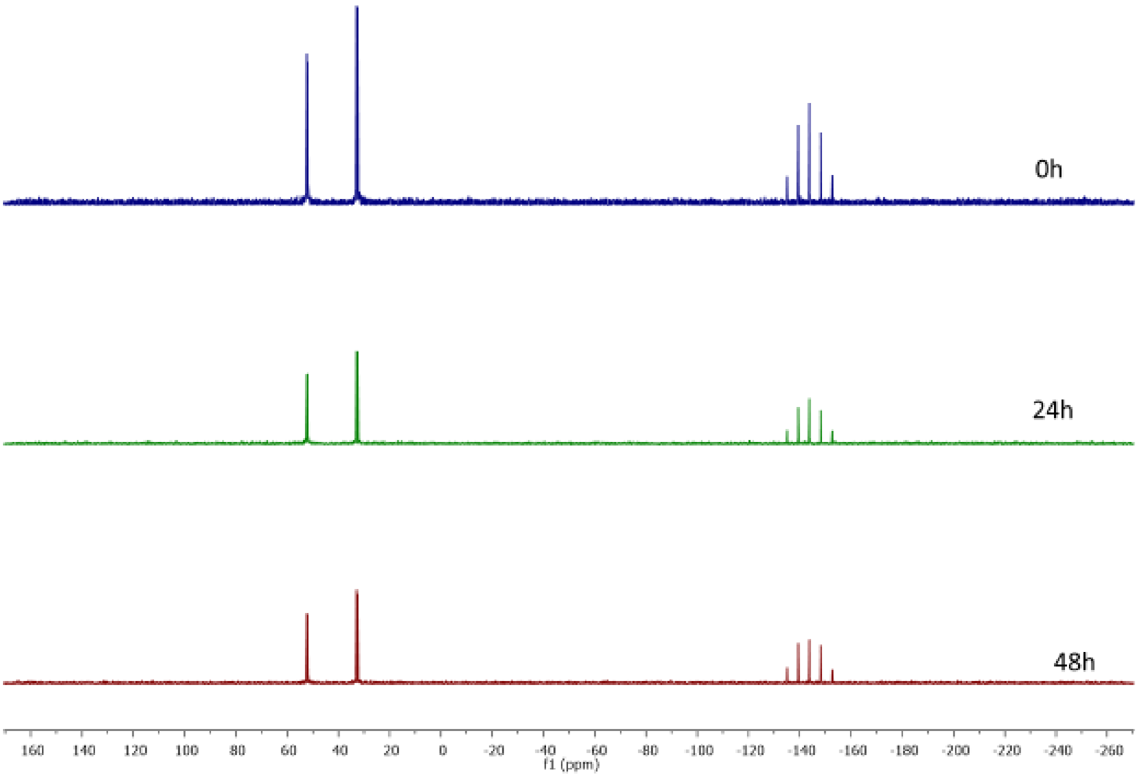

Figure S20 – $^{31}P\{^{1}H\}$ spectra of complex 2, in DMSO/D_2O at different times.

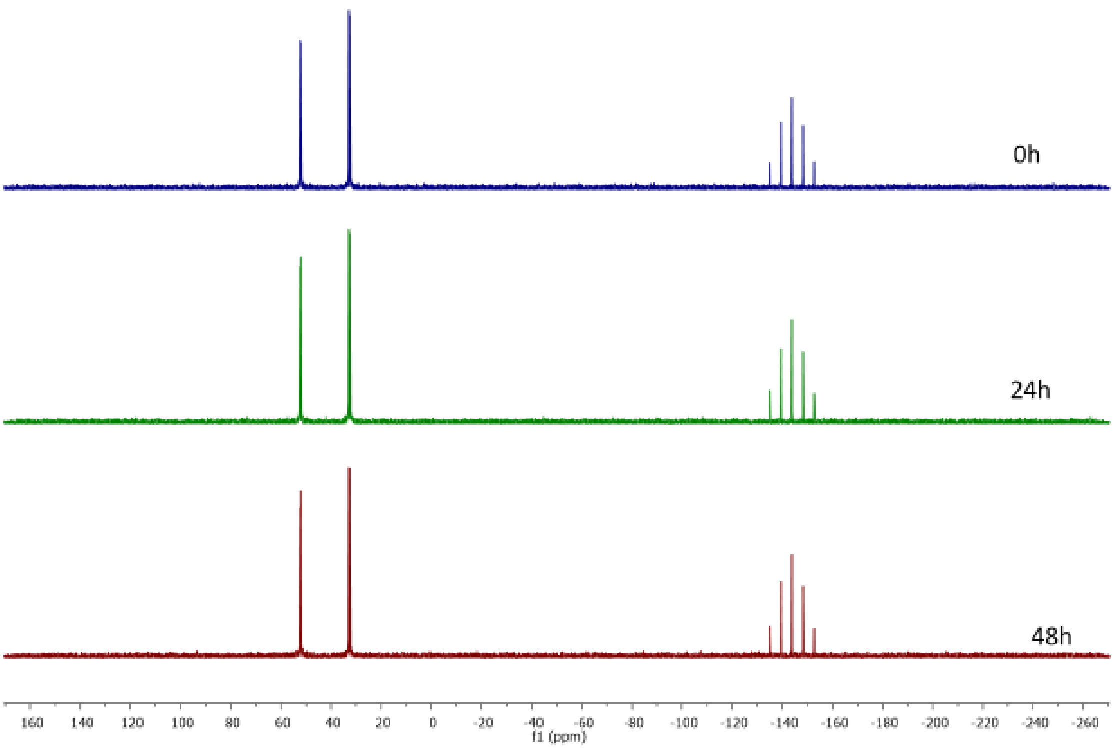

Figure S21 – $^{31}P\{^{1}H\}$ spectra of complex 3, in DMSO/D_2O at different times.

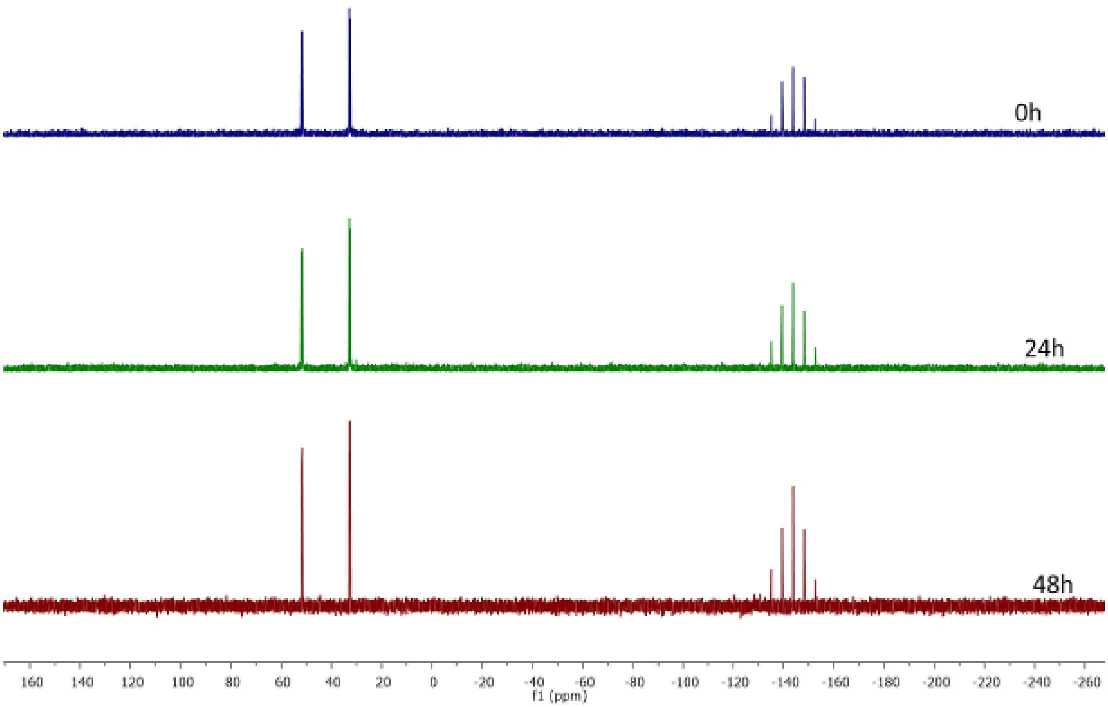

Figure S22 – $^{31}P\{^{1}H\}$ spectra of complex 4, in DMSO/D_2O at different times.

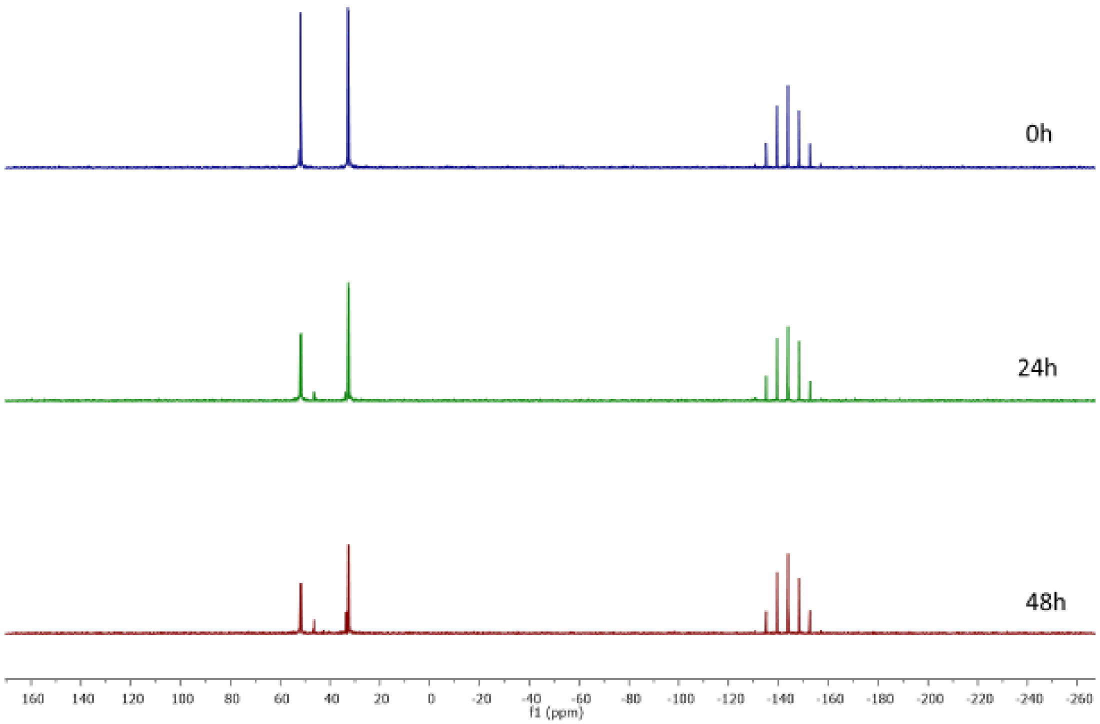

Figure S23 – $^{31}P\{^{1}H\}$ spectra of complex 1, in DMSO/DMEM/D_2O (90:10, *v*/*v*) at different times.

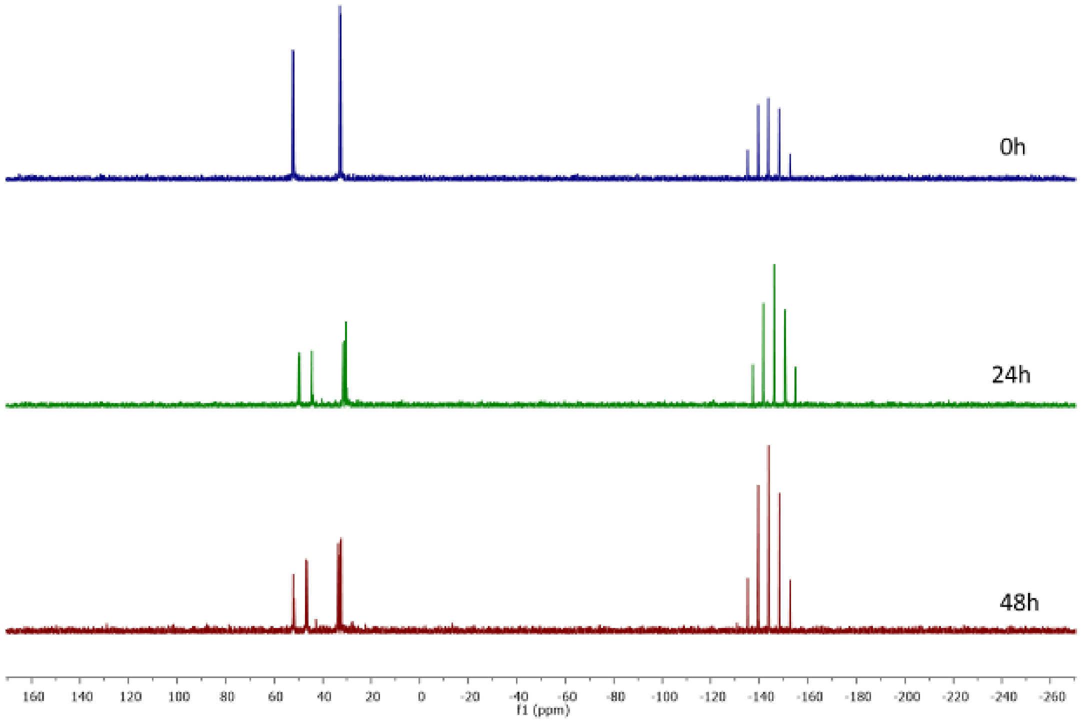

Figure S24 – $^{31}P\{^{1}H\}$ spectra of complex 2, in DMSO/DMEM/D_2O (90:10, *v*/*v*) at different times.

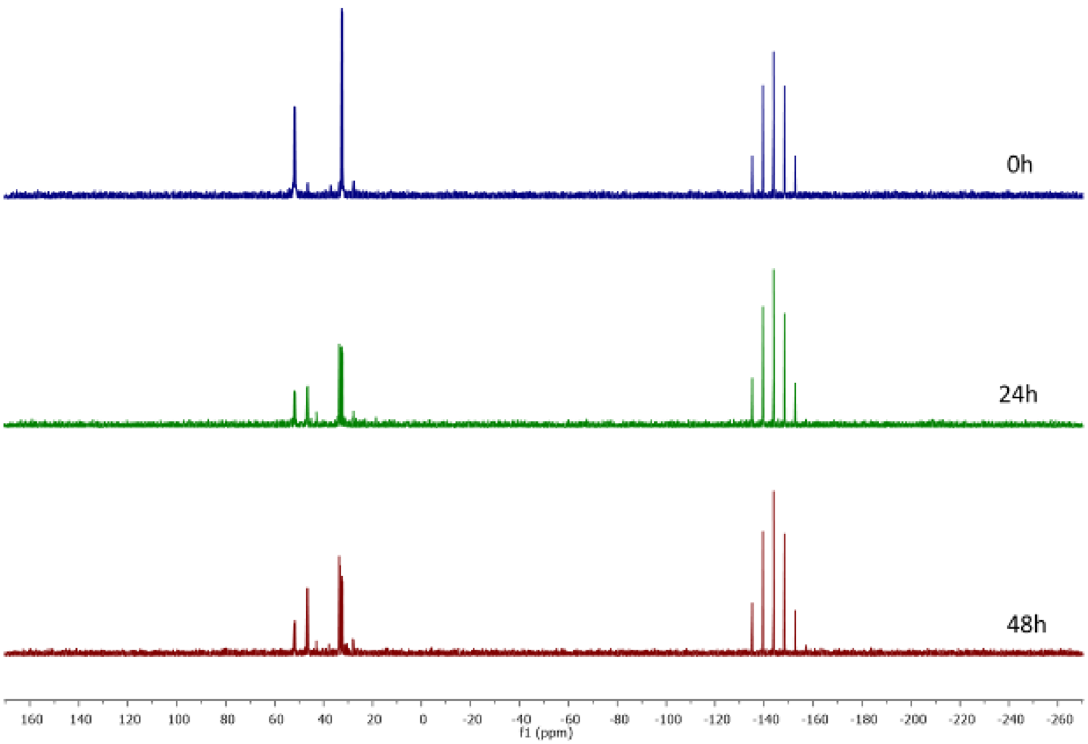

Figure S25 – $^{31}P\{^{1}H\}$ spectra of complex 3, in DMSO/DMEM/D_2O (90:10, *v*/*v*) at different times.

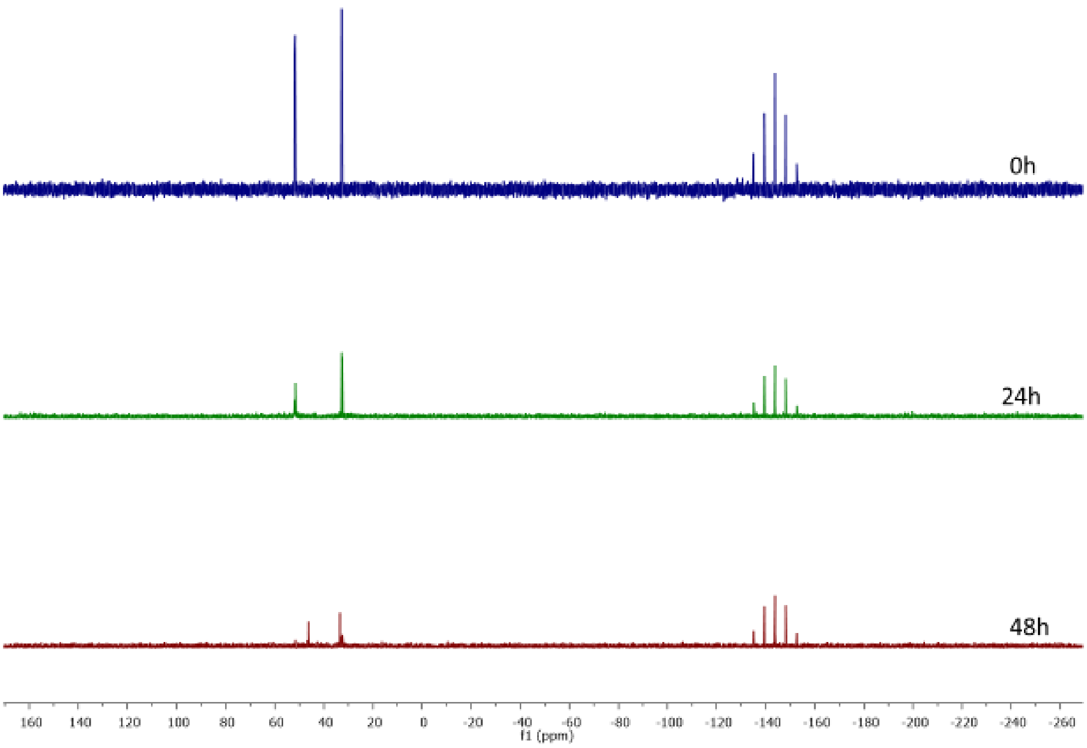

Figure S26 – $^{31}P\{^{1}H\}$ spectra of complex 4, in DMSO/DMEM/D_2O (90:10, *v*/*v*) at different times.

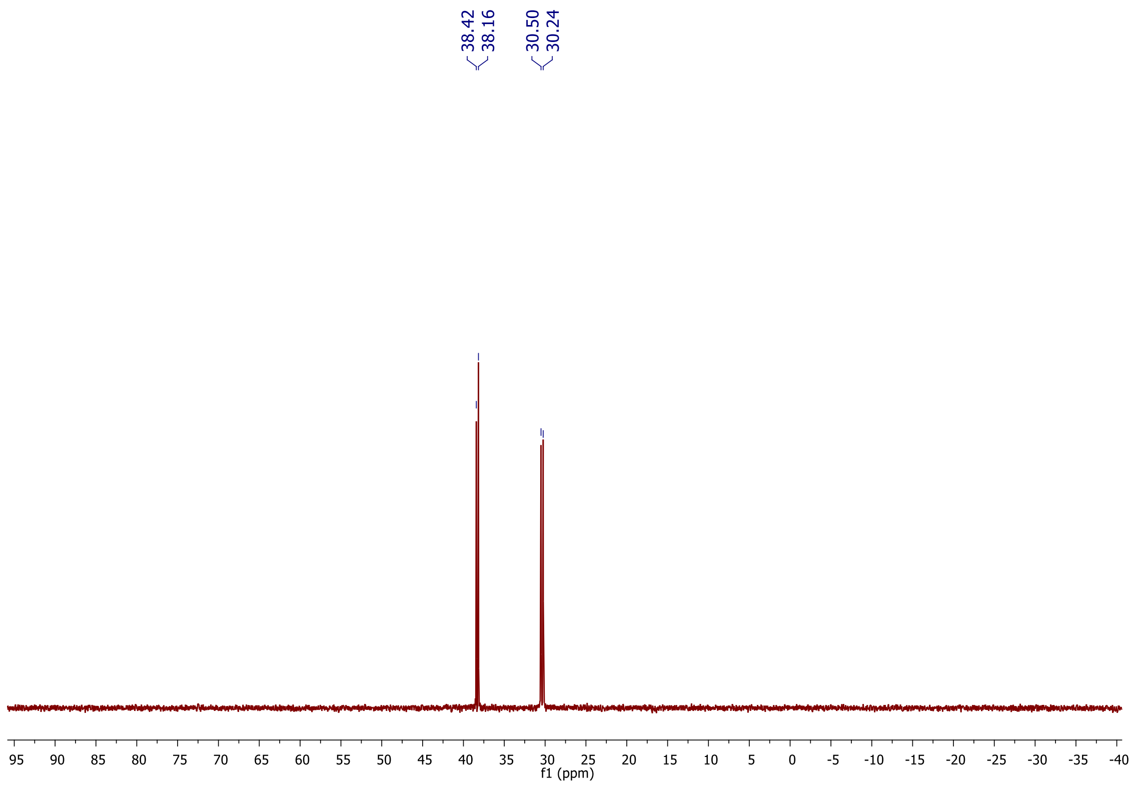

Figure S27 - $^{31}P\{^{1}H\}$ NMR spectrum of precursor in CH_2Cl_2/D_2O at 298K.

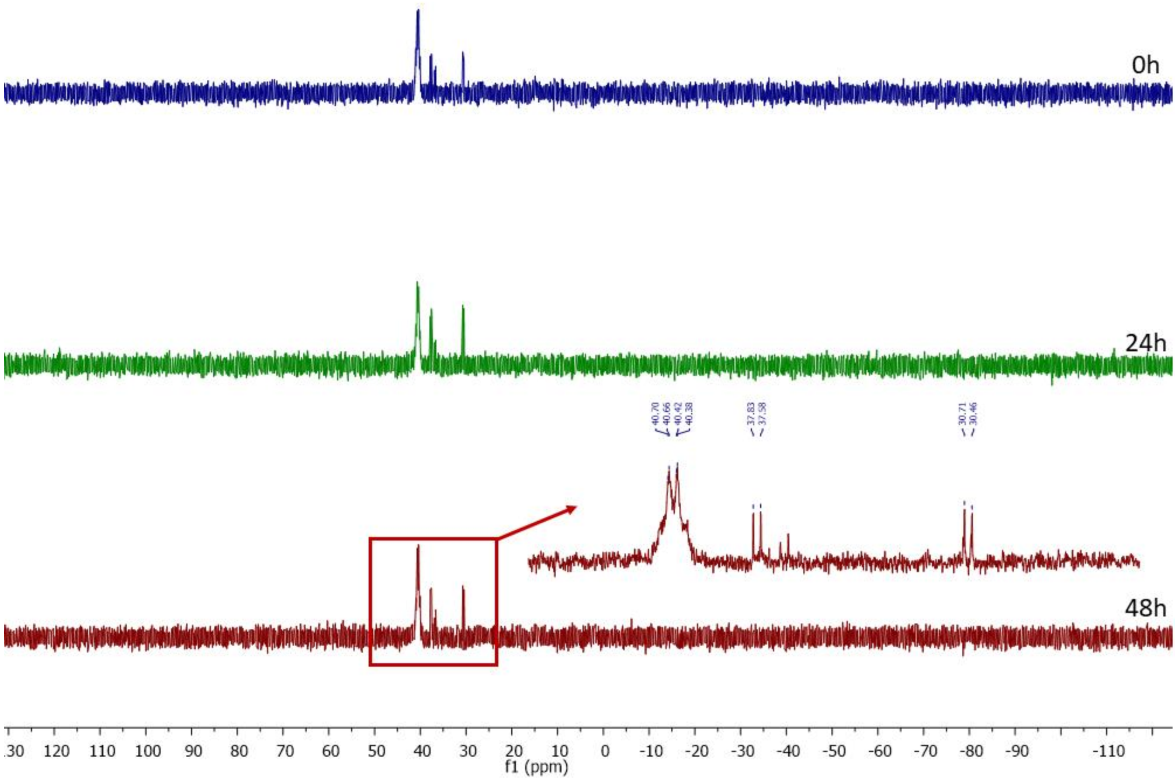

Figure S28 - $^{31}P\{^{1}H\}$ spectra of precursor, in $DMSO/D_2O$ at different times.

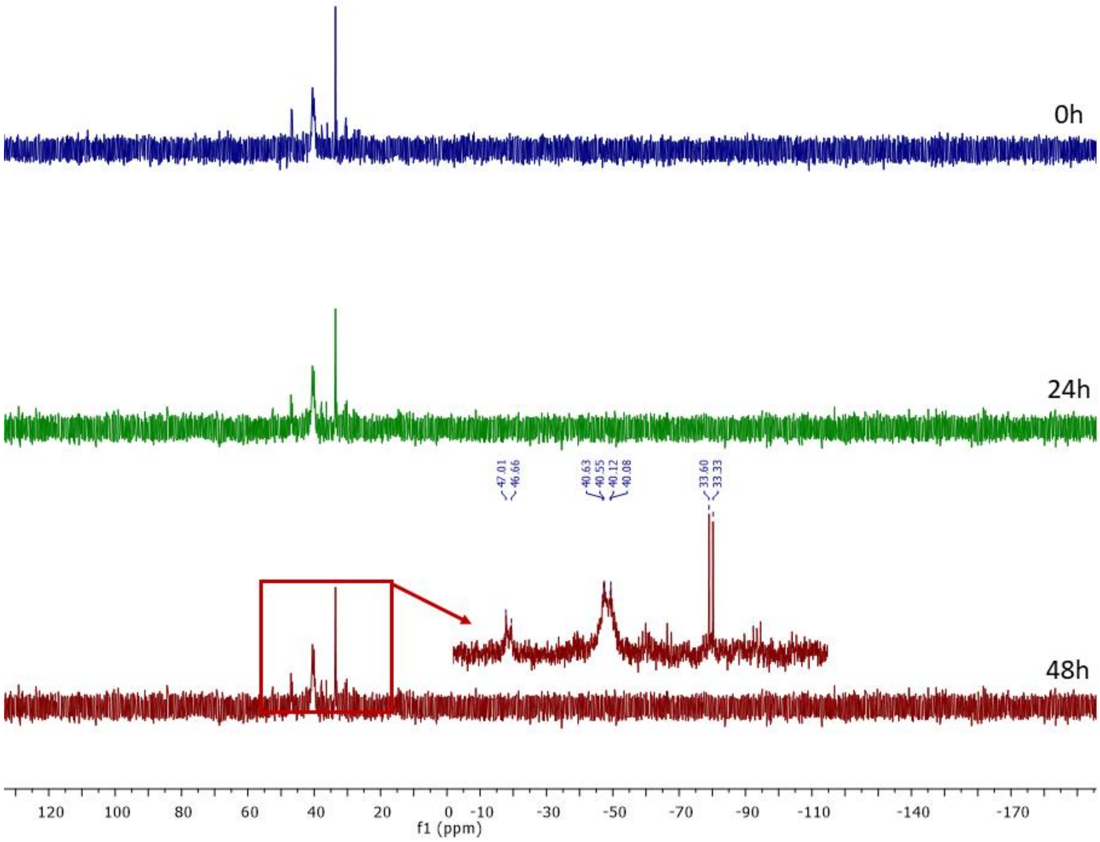

Figure S29 - $^{31}P\{^{1}H\}$ spectra of precursor, in DMSO/DMEM/D_2O (90:10, v/v) at different times.

Crystallographic data and details of the refinement of the compounds

Table S2 - X-Ray crystallographic data collection and refinement parameters for complex 1.

**Compound**	**1**
Formula	$C_{50}H_{44}F_6N_3O_2P_3Ru$
$D_{calc.}$/ g cm^{-3}	1.384
m/mm^{-1}	4.040
Formula Weight	1026.86
Colour	red
Shape	plate
Size/mm^3	0.09×0.06×0.03
T/K	209.99(10)
Crystal System	monoclinic
Flack Parameter	-0.020(4)
Hooft Parameter	-0.024(3)
Space Group	$P2_1$
a/Å	10.10490(10)
b/Å	22.6259(2)
c/Å	10.90260(10)
a/°	90
b/°	98.6690(10)
g/°	90
V/Å^3	2464.21(4)
Z	2
Z'	1
Wavelength/Å	1.54184
Radiation type	Cu K_a
Q_{min}/°	4.426
Q_{max}/°	70.076
Measured Refl.	29007
Independent Refl.	8671
Reflections with I > 2(I)	8398
R_{int}	0.0385
Parameters	585
Restraints	0
Largest Peak	0.639
Deepest Hole	-0.330
GooF	1.036
wR_2 (all data)	0.0695
wR_2	0.0678
R_1 (all data)	0.0298
R_1	0.0282

Table S3 - Main distances [Å] and angles [°] angles for the complex 1.

**Fragment**	**(1)**
Ru1-O1	2.226(3)
Ru1-O2	2.130(3)
Ru1-P1	2.2424(10)
Ru1-P2	2.3131(10)
Ru1-N3	2.068(3)
Ru1-N2	2.111(3)
P1-Ru1-P2	89.76(3)
N2-Ru1-N3	78.34(14)
O1-Ru1-O2	60.02(11)

DNA interaction

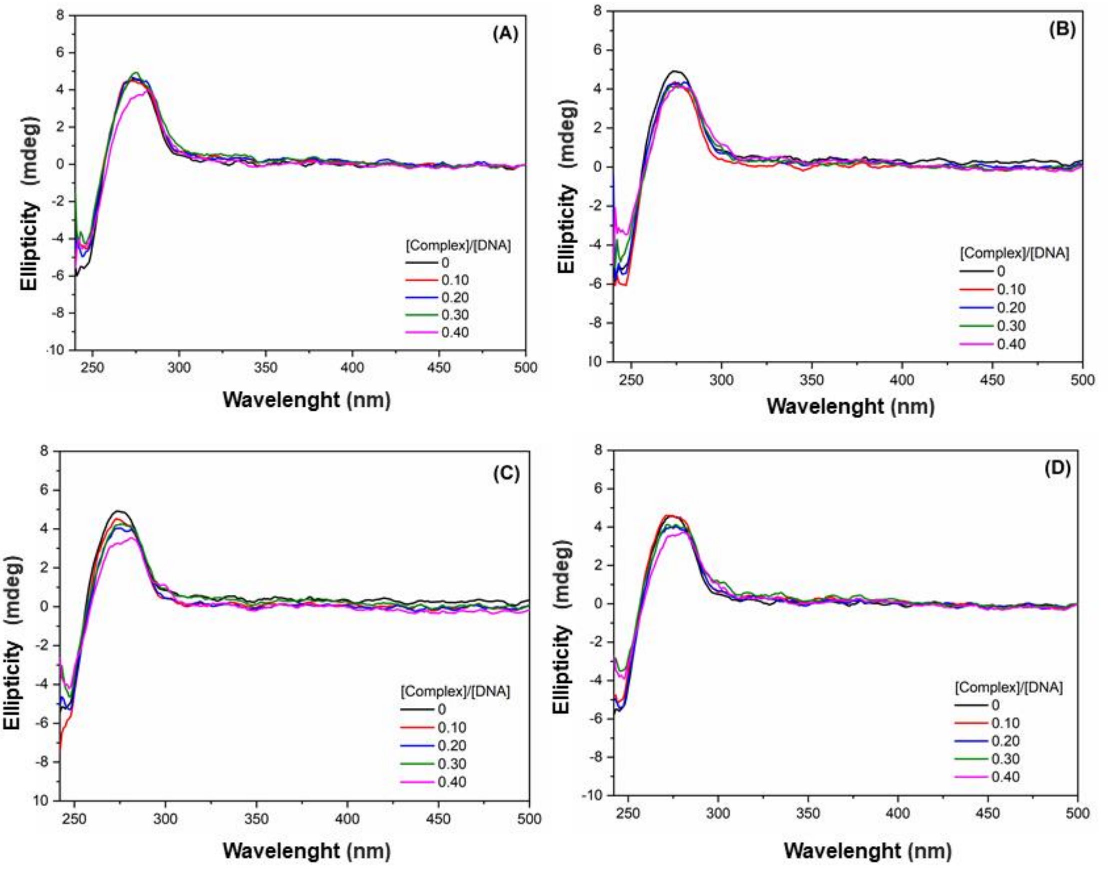

Figure S30 - CD spectra of ct-DNA (100 μM) in the absence and presence of complexes (A) 1, (B) 2, (C) 3 and (D) 4 at different complex-to-DNA molar ratios, in Tris-HCl buffer (pH 7.4).

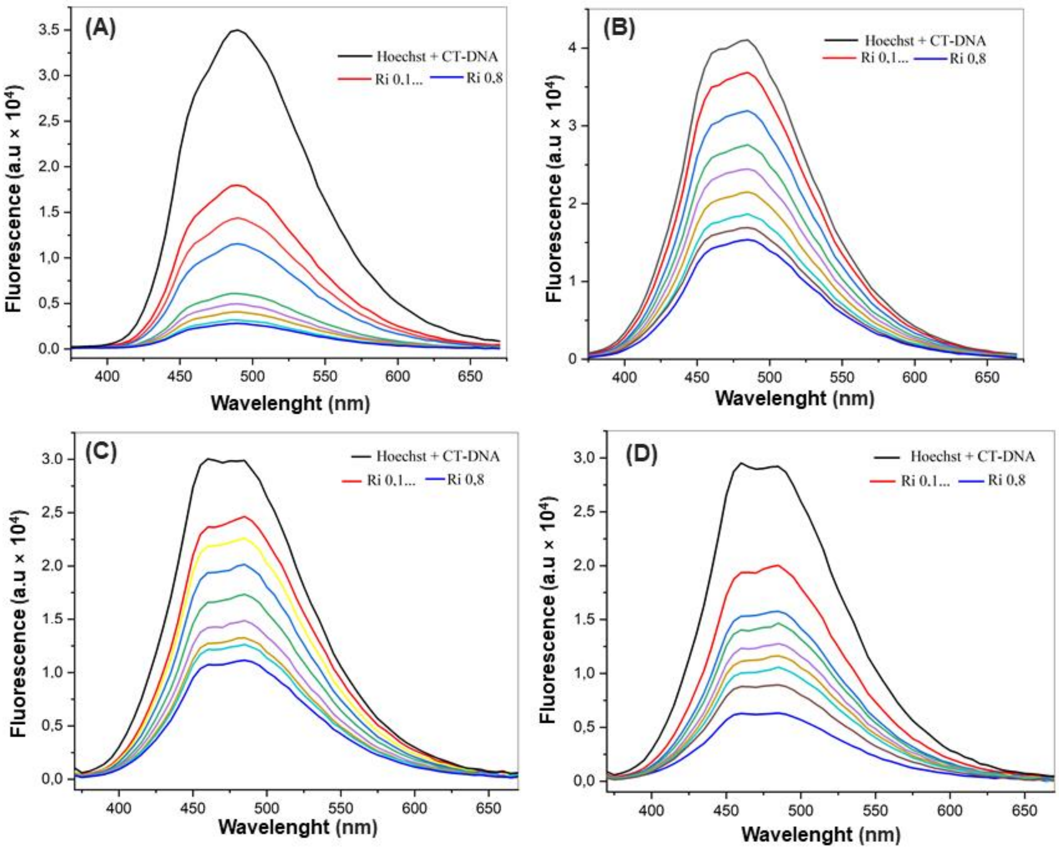

Figure S31 - Fluorescence quenching spectra of Hoechst (2.5 μM)/DNA (125 μM) in the absence and presence of complexes (A) 1, (B) 2, (C) 3 and (D) 4, at different [compound]/[DNA] ratios (0; 0.1; 0.2; 0.3; 0.4; 0.5; 0.6; 0.7; 0.8) with an excitation wavelength at 340 nm at 310 K in a Tris-HCl buffer.

Morphologic assay

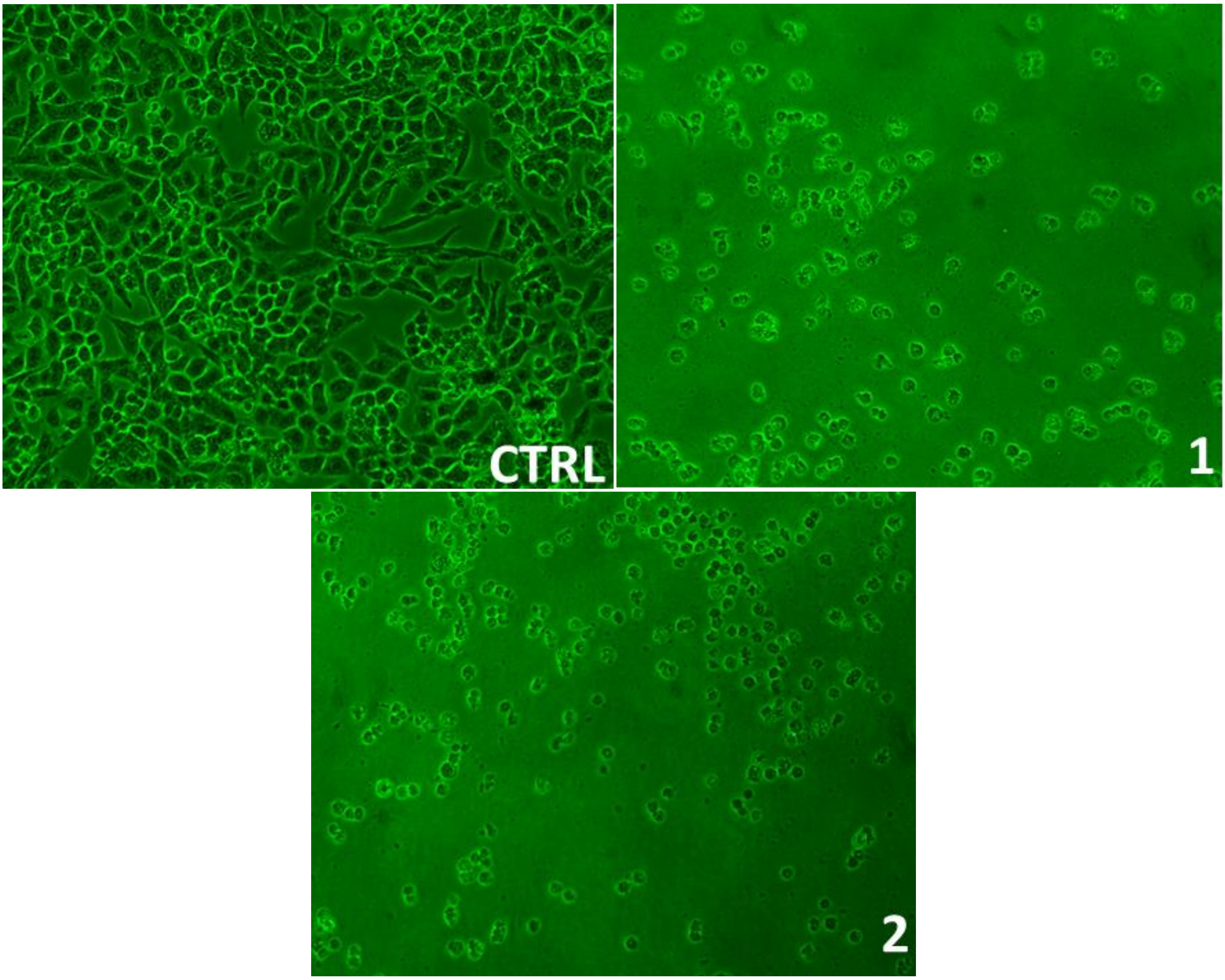

Figure S32 – Amplification of microscopy images showing the cellular morphology of A2780 ovarian cancer cells after 48h upon treatment with **1** and **2** at 2 × IC_{50} concentrations. DMSO was used as negative control. The images were taken using a NIKON ECLIPSE TS100 microscope and recorded at 10× zoom.

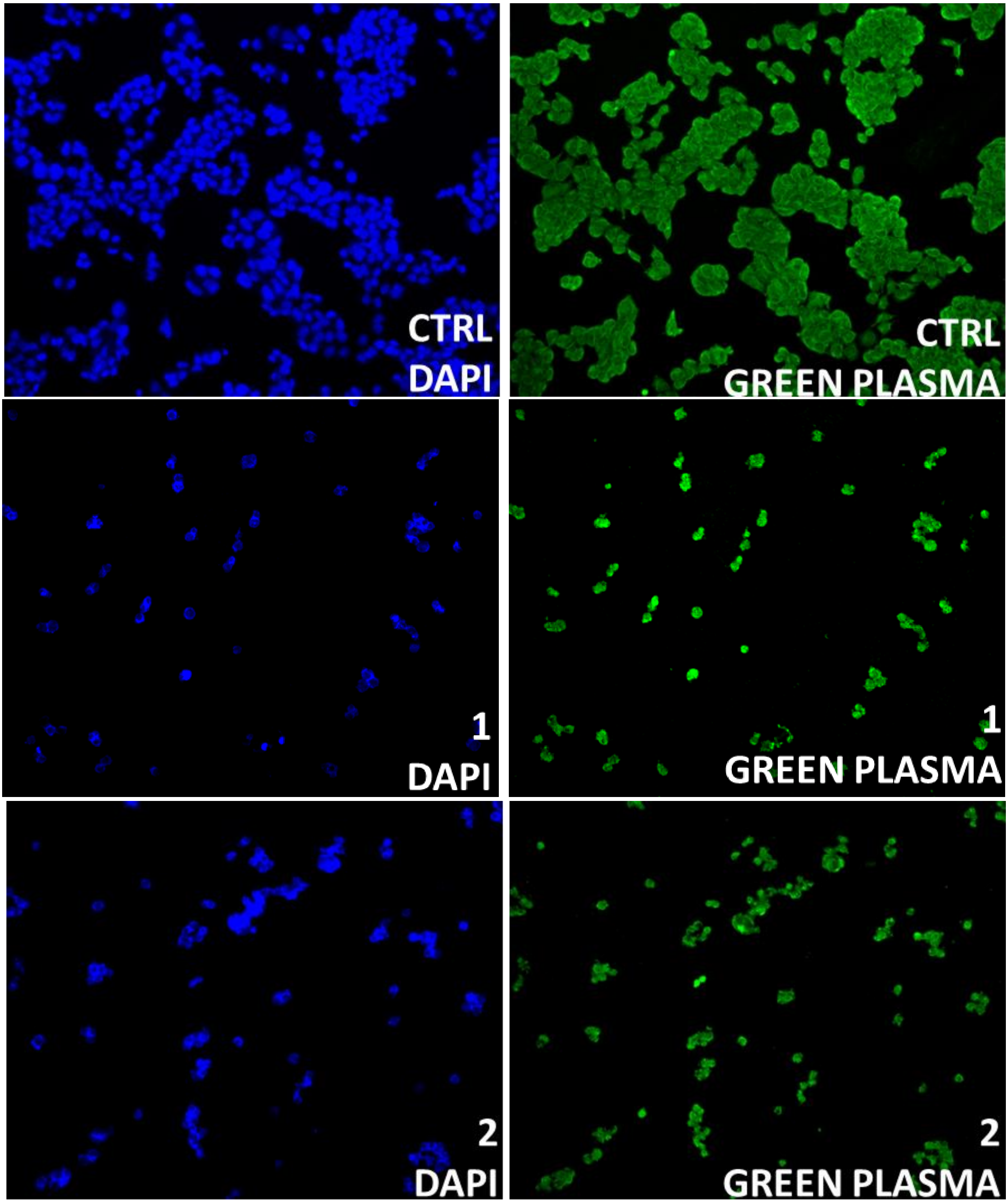

Figure S33 – Amplification of fluorescence microscopy images showing the cellular morphology of A2780 ovarian cancer cells using fluorescence with Green Plasma and DAPI markers after 48h upon treatment with **1** and **2** at 2 × IC_{50} concentrations. DMSO was used as negative control. The images were taken using a CELENA® microscope and recorded at 10× zoom.

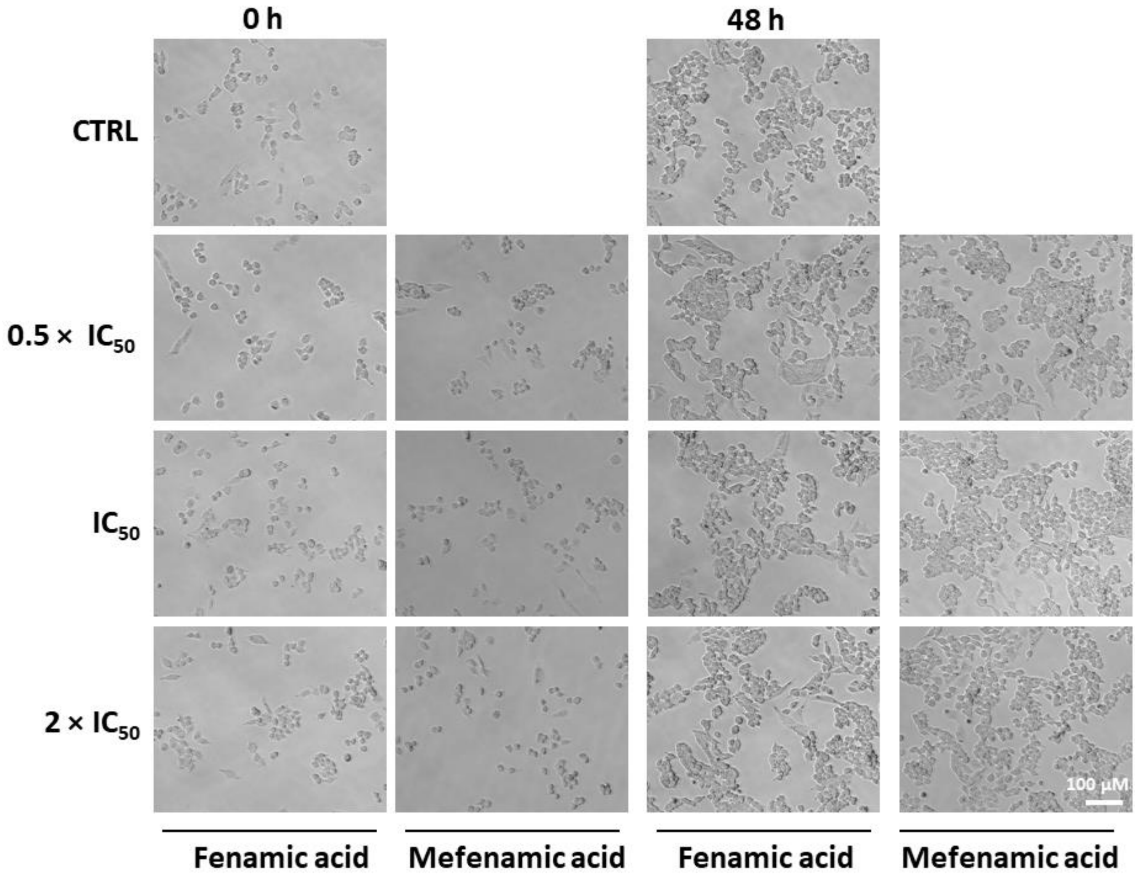

Figure S34 - Microscopy images showing the cellular morphology of A2780 ovarian cancer cells after 0 and 48h upon treatment with **fenamic acid** and **mefenamic acid** ligands at 0.5 × $IC_{50,}$ IC_{50} and 2 × IC_{50} concentrations of complexes **1** and **2**. DMSO was used as negative control. The images were taken using a CELENA® microscope and recorded at 10× zoom.

Cellular uptake by HR-CS GFAAS

Table S4 - Graphite furnace program for ruthenium AAS measurements

Step	Name	Temp. (°C)	Ramp. [°C s^{-1}]	Hold [s]
1	Drying	90	10	40
2	Drying	105	7	30
3	Drying	120	15	20
4	Pyrolysis	500	50	30
5	Pyrolysis	900	200	20
6	Gas adaption	900	0	6
7	Atomize	2450	maximum	4
8	Clean	2600	1000	6

Table S5 - Instrumental parameters used in the determination of Ru in cell suspension by HR-CS GFAAS.

Wavelength (nm)	349.8945
Read time (s)	8
Integration mode of absorbance sinal	Area
Evaluation pixels	3
Purge gas	Argon
Gas flow during atomization	Stop
Working range (ng)	0- 1.5

Table S6 - Percentage of Ru uptake in the cells determined by HR-CS GFAAS.

Sample	[Ru] (ng)	Initial concentration (pg)	Uptake percentage (%)
1	20,8 ± 3,2	571,3 ± 8,9	3,6
2	26,0 ± 1,9	556,0 ± 9,9	4,5

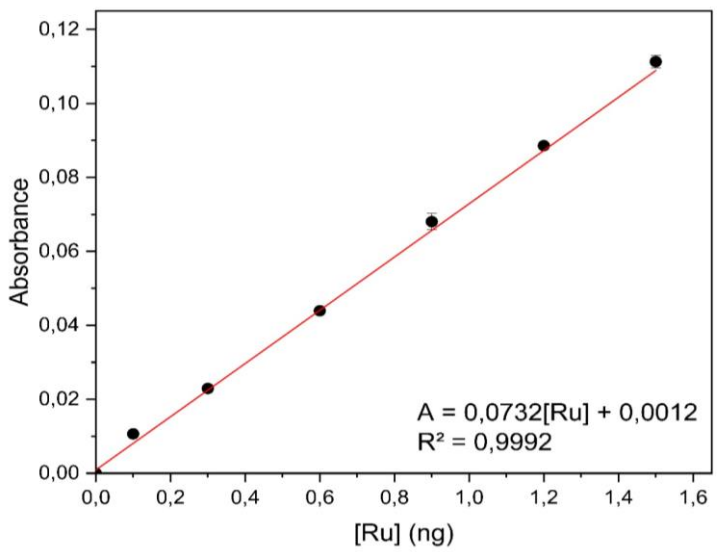

Figure S35 - Calibration curve obtained for Ru determination by HR-CS GFAAS.
